# Randomized trial of two doses of vitamin D_3_ in preterm infants <32 weeks: Dose impact on achieving desired serum 25(OH)D_3_ in a NICU population

**DOI:** 10.1371/journal.pone.0185950

**Published:** 2017-10-10

**Authors:** Ann Anderson-Berry, Melissa Thoene, Julie Wagner, Elizabeth Lyden, Glenville Jones, Martin Kaufmann, Matthew Van Ormer, Corrine Hanson

**Affiliations:** 1 Department of Pediatrics, University of Nebraska Medical Center, Omaha, Nebraska, United States of America; 2 Neonatal Intensive Care Unit, The Nebraska Medical Center, Omaha, Nebraska, United States of America; 3 CHI Health, Omaha, Nebraska, United States of America; 4 College of Public Health, University of Nebraska Medical Center, Omaha, Nebraska, United States of America; 5 Department of Biomedical and Molecular Sciences, Queen’s University, Kingston, Ontario, Canada; 6 Division of Medical Nutrition Education, School of Allied Health Professions, University of Nebraska Medical Center, Omaha, Nebraska, United States of America; TNO, NETHERLANDS

## Abstract

**Background:**

Recommendations for vitamin D supplementation for preterm infants span a wide range of doses. Response to vitamin D supplementation and impact on outcomes in preterm infants is not well understood.

**Objective:**

Evaluate serum 25(OH)D_3_ concentration changes after 4 weeks in response to two different doses of vitamin D_3_ supplementation in a population of premature infants and quantify the impact on NICU outcomes.

**Design:**

32 infants born at 24–32 weeks gestation were prospectively randomized to receive 400 or 800 IU/day vitamin D_3_ supplementation. Serum 25(OH)D_3_ levels were measured every 4 weeks. The Wilcoxon signed rank test was used to compare serum levels of 25(OH)D_3_ at 4 weeks and each subsequent time point. A p-value of <0.05 was considered statistically significant.

**Results:**

Serum 25(OH)D_3_ levels at birth were 41.9 and 42.9 nmol/l for infants in the 400 IU group and 800 IU group, respectively (p = 0.86). Cord 25(OH)D_3_ concentrations significantly correlated with gestational age (r = 0.40, p = 0.04). After 4 weeks of D_3_ supplementation, median 25(OH)D_3_ levels increased in both groups (84.6vs. 105.3 nmol/l for 400 vs. 800 IU/day respectively, with significantly more improvement in the higher dose (p = 0.048). Infants in the 400 IU group were significantly more likely to have dual energy x-ray absorptiometry (DEXA) bone density measurements <10 percentile (56% vs 16%, p = 0.04).

**Conclusions:**

Improvement in 25(OH)D_3_ levels at 4 weeks, bone density, and trends towards improvement in linear growth support consideration of a daily dose of 800 IU of vitamin D for infants <32 weeks cared for in the NICU.

## Background

Vitamin D status has impact on current and future health in children and neonates and has documented impact in most organ systems in the body, making it a critical area of research [1]. Many aspects of clinical application of vitamin D supplementation in the smallest preterm infants need additional supportive evidence. Vitamin D supplementation for preterm and term neonates is currently recommended by many groups including the American Academy of Pediatrics (AAP), the Institute of Medicine (IOM), the Endocrine Society, and the European Society for Paediatric Gastroenterology, Hepatology and Nutrition (ESPGHAN) [2]. However, significant differences remain in the recommended target goals for 25(OH)D levels and in the recommended doses for specific neonatal populations from these expert bodies (Table 1). The IOM recommends a 25(OH)D level of 50 nmol/l based on bone health and mineralization. This recommendation is based, in part, off of work by Priemel et al evaluating bone mineralization defects and vitamin D deficiency in iliac crest bone of adults [3]. The authors of this paper however, conclude that doses of supplementation of vitamin D should ensure 25(OH)D levels of 75 nmol/l to maintain skeletal health. There is an obvious lack of substantial data in infancy with regards to bone health and ideal supplementation. Additionally, many recommendations are specifically for term neonates and do not apply to preterm, very low birth weight (VLBW) or extremely low birth weight (ELBW) infants. The AAP and ESPGHAN statements that do provide recommended intakes for preterm infants differ, and are based on limited prospective randomized controlled trial evidence [2–4]. The Endocrine Society also recommends a target level of >75 nmol/l and ESPGHAN an even higher target of >80 nmol/l.

**Table 1:** Recommendations for vitamin D supplementation.

Recommending Body	Patient Age	Recommended Supplementation	Recommended Target Serum Level	Comments	Year of Publication
**American Academy of Pediatrics—Section of Breastfeeding and Committee on Nutrition**	Healthy infants birth– 12 months of age	400 IU daily	>50 nmol/l (targeting bone health)	*Begin soon after birth, in the first few days of life. *All breastfeed and partially breastfed infants. *Formula fed infants taking <1 liter of formula	2008[5]
**AAP—committee on nutrition**	Preterm Infants VLBW	200–400 IU	>50 nmol/l (targeting bone health)	Discharge vitamin D recommended for breastfed infants 400 IU, for formula fed 200–400 IU	2013[6]
**AAP—committee on nutrition**	Preterm infants>1500g	400–1000 IU	>50 nmol/l (targeting bone health)	Tolerating Full Enteral Feeds	2013[6]
**World Health Organization**	Preterm Infants	400–1000 IU		Low and Middle-income countries	WHO[7]
**Institute of Medicine**	Infants 0–6 months	400 IU	>50 nmol/l (targeting bone health)	Under assumption of minimal sunlight	2011[8]
**ESPHAGAN**	Preterm Infants	800–1000 IU	>80 nmol/L	Stable Growing, 1000 to 1800 grams	2010[4]
**Pediatric Endocrine Society**	Breastfed Infants, or those taking <1Lformula/day	400 IU	>50 nmol/l (desire additional studies to determine if >80 nmol/L is optimal)	800 IU for high risk populations i.e. preterm infants	2008[2]
**Endocrine Society**	Healthy Infants 0–1 year	400–1000 IU	>75 nmol/l (for non-skeletal benefits)	0–1 vitamin D deficient (<50 nmol/l) 2000 IU/D for 6 weeks followed by maintenance dosing	2011[9]

Very little data is available regarding response to vitamin D supplementation in the smallest and youngest infants (<1200 grams at birth), a population that might see the most benefit from normalized vitamin D status. Supplementation during NICU hospitalization with 800 IU in a cohort of infants <1500 grams showed no safety concerns with all infants having levels above 25 nmol/l but 21% of infants still insufficient at 36 weeks CGA [10]. Another trial evaluating placebo, 200 IU and 800 IU daily vitamin D supplement evaluated levels at 36 weeks and days of respiratory support, showing prevention of vitamin D deficiency with the 800 IU dose, and an improvement in serum levels with the 200 IU dose as compared to placebo, but no difference in days alive or in respiratory support [11].

Historically, levels of 25(OH)D greater than 50 nmol/l have been associated with prevention of rickets in infants and children, a disease of extreme nutritional deficiency. As the role of vitamin D in immune function, respiratory health, allergy and atopic disease, and many other tissue and organ functions becomes apparent, adjustments have been suggested in optimal serum level of 25(OH)D [12–14]. Recent literature from adult, pediatric and neonatal populations demonstrate improved outcomes such as normalization of PTH at levels >75 nmol/l [15–17].

It is likely that preterm infants will have a compromised vitamin D status at birth. Many studies in the United States and abroad have documented maternal status to be suboptimal and have called for national programs to improve maternal vitamin D status with increased supplementation dose and frequency [18–26]. The fetus relies solely on maternal stores for vitamin D in utero, and typically has cord blood levels approximately 50–75% of the maternal value [6,27,28]. Bodnar showed as few as 4.1% of black and 37.3% of term white infants having adequate (>80 nmol/l) levels at delivery, while McCarthy showed 78% of infants to have lower than optimal levels [18,29]. Importantly, several studies have documented an association with decreasing 25(OH)D levels and lower gestational age at delivery allowing for a situation where preterm infants are a highly vulnerable population at birth with regards to their vitamin D status [15,28,30].

Vitamin D intake during the first year of life from milk or formula diet and supplemental foods has not been shown to consistently equal 400 IU/day, the amount recommended by the AAP or the IOM for term infants [31,32]. Preterm infants have a smaller size, leading to decreased volume intake, and often have a compromised nutritional status during the initial hospitalization and are even less likely to get adequate vitamin D intake from diet [15].

Our objective with this double blinded, randomized controlled trial was to evaluate the effects of two different doses (400 IU vs 800 IU) of vitamin D_3_ on a preterm patient population for the duration of Neonatal Intensive Care Unit (NICU) hospitalization with respect to improvement in percentage of infants with 25(OH)D levels >75 nmol/l, growth, PTH normalization, and bone density as measured by global (DEXA) measurements. Additionally, we will evaluate response to supplementation in the subset of infants weighing <1200 grams as there is minimal information available regarding response to vitamin D supplementation in this specific high-risk group.

## Methods

**Fig 1:**
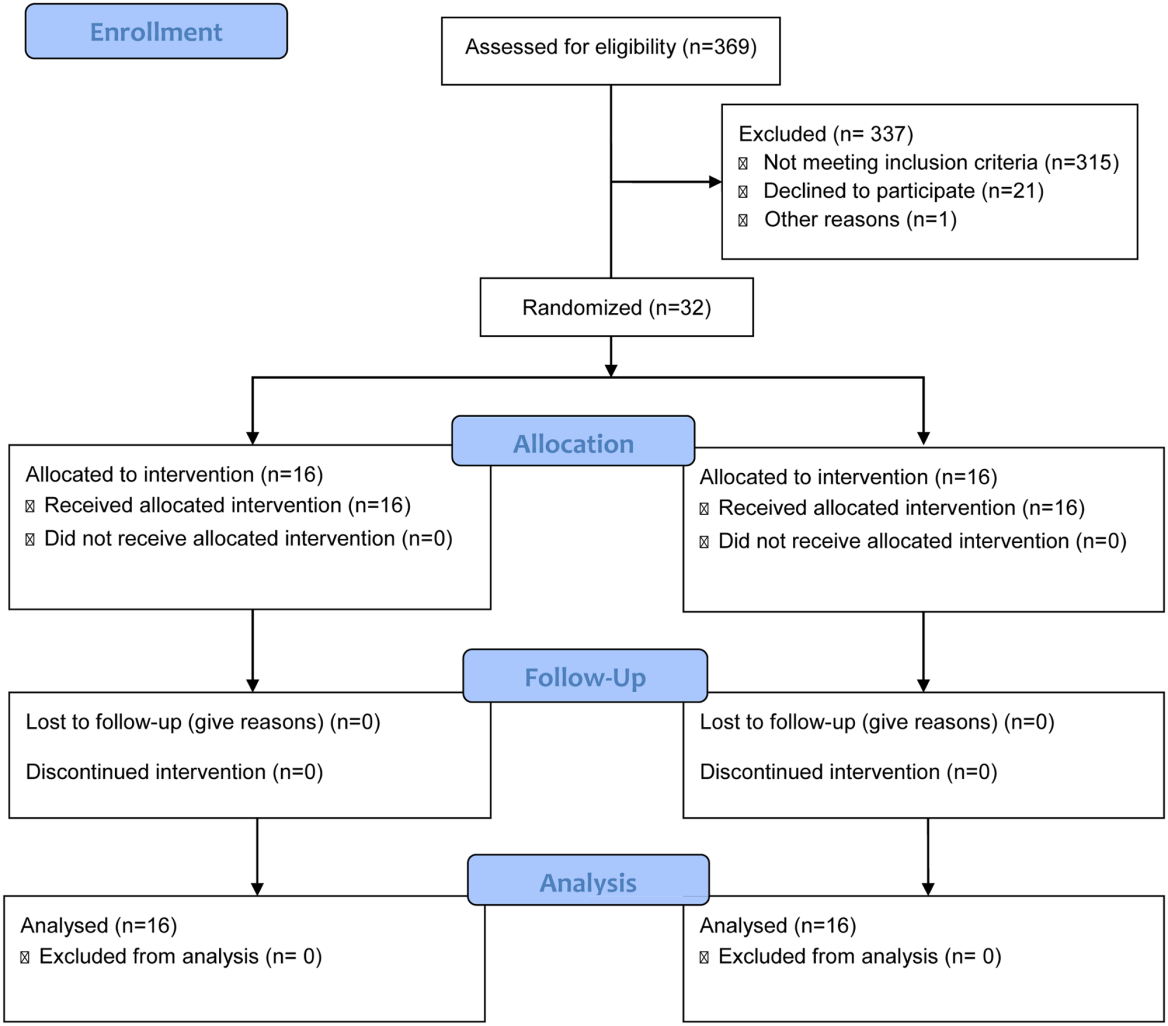
Flow chart of infant screening and enrollment.

Institutional Review Board permission was obtained (final version– 3, approved March 1, 2012) to enroll with parental consent from parents aged 19 or greater, patients <32 weeks gestational age to be enrolled in the randomized controlled trial in a Midwestern academic medical center Newborn Intensive Care Unit. The study statistician generated a randomization sequence stratified by race (white and non-white) using SAS software and the study pharmacist randomized each infant. Infants were randomized to receive either 400 IU or 800 IU of vitamin D_3_ enterally with the initiation of enteral feedings in addition to parenteral MVI while on parenteral nutrition and enteral vitamin D from breast milk and human milk fortifier or preterm formula. The study vitamin D was delivered in a brown oral syringe (to protect the product from light) and the product was identical in color, volume and smell regardless of dose. The screening and flow of infant enrollment is shown in Fig 1. All vitamin D formulations were prepared and dispensed by a research pharmacist who was independent of the study. Investigators and NICU staff were blinded to subject group assignment. Serum 25(OH)D_3_ levels were measured every 4 weeks simultaneously in triplicate using a novel, very sensitive LC-MS/MS-based method involving derivatization with DMEQ-TAD in the Department of Biomedical and Molecular Sciences at Queens University, Ontario, Canada. Serum iPTH was measured using The Access Intact PTH assay (Beckman-Coulter Inc, Fullerton, CA, USA) [33]. Serum calcium was measured using the SYNCHRON^®^ System (Beckman-Coulter Inc, Fullerton, CA, USA). At-term corrected gestational age (40 weeks +/- 4 weeks) bone density was measured by total body DEXA scan (Discovery A (SIN85958) Hologic, Software v.13.3). Premature infant bone density was evaluated based on curves published by Rigo et al [34]. Exclusion criteria included infants with congenital abnormalities, gastro-intestinal, liver, or kidney disease, inborn errors of metabolism, parathyroid disease, disorders of calcium metabolism, and infants receiving seizure medication or steroids. The study was registered at www.clinicaltrials.gov (NCT01469650).

### Statistical analysis

A sample size of 32 infants (16 per group) was needed to achieve 80% power to detect a difference after 4 weeks of intervention of 7.2 ng/ml (18 nmol/L) between the null hypothesis that both group means are 23.1 ng/ml (57.7 nmol/L) 25(OH)D and the alternative hypothesis that the mean of the 800 IU group is 30.3 ng/ml (75.6 nmol/L) 25(OH)D with known within-group standard deviation of 7.0 ng/ml (17.5 nmol/L) and an α = 0.05. All data were analyzed following the intention-to-treat paradigm, in which infants were analyzed according to their randomized assignment. The Mann-Whitney test was used to compare continuous data between the dose groups and the Wilcoxon signed rank test was used for comparisons between time points. Fisher’s exact test was used to compare categorical data between the dose groups. Associations between continuous variables were assessed with the Spearman correlation coefficient. All statistical analysis were performed utilizing SAS Version 9.4 (SAS Institute Inc., Cary, NC). A p-value of <0.05 was considered statistically significant.

## Results

### Baseline characteristics

Thirty-two infants were enrolled in the study (16 per group) and were included in the final analysis. 38% of the total study population was classified as non-white and this was equal in both dosing groups. Infants in the 400 IU dosing group were 43% female, and infants in the 800 IU group were 50% female, a non-significant difference. There were no significant differences between the two groups at birth. Gestational age, birth anthropometrics and in hospital growth are presented in Table 2.

**Table 2:** Gestational age, birth anthropometrics and in hospital growth by vitamin D_3_ supplementation group.

Variable	400 IU			800 IU			p-value
	N	Median	IQR	N	Median	IQR	
**Birth weight, grams**	16	1405.5	270	16	1392.5	632	1.00
**Birth weight %ile**	16	33	30	16	39.5	39	0.33
**Gestational Age at Delivery**	16	31	2	16	30.35	5.35	0.75
**Birth length, cm**	16	39	5.5	16	39.5	10.1	0.94
**Birth length %ile**	16	15	24	16	24.5	36.5	0.36
**Birth head circumference, cm**	16	27.5	2	16	27.5	4.15	0.66
**Birth HC %ile**	16	20	51	16	39	40.5	0.71
**Weight at 36 weeks, grams**	14	2378.5	393	12	2542	472.5	0.23
**Weight %ile 36 weeks**	14	18	20	12	27.5	31	0.14
**Length at 36 weeks, cm**	14	43	2.2	12	45.75	3.65	0.09
**Length %ile 36 weeks**	14	11	15	12	28	45	0.06
**HC at 36 weeks, cm**	14	32.1	3.5	12	33.25	2.05	0.75
**HC %ile 36 weeks**	14	30.5	62	12	44	29.5	0.52

### 25OHD changes by vitamin D dose

Cord blood 25(OH)D_3_ levels were significantly correlated with gestational age (r = 0.40, p = 0.04), but had no correlation with sex or race. Concentration of 25(OH)D_2_ were not detectable in any of the samples; therefore, all results presented are for serum 25(OH)D_3_ concentrations. After 4 weeks of vitamin D_3_ supplementation in addition to dietary vitamin D intake, median 25(OH)D_3_ levels increased in both groups (84.6 vs. 105.3 nmol/l for 400 vs. 800 IU/day respectively), with significantly greater improvement in the higher dose group (p = 0.048). After 8 weeks of D_3_ supplementation, median 25(OH)D_3_ levels increased in both groups (135 vs. 164 nmol/l for 400 vs. 800 IU/day respectively, p = 0.3) (Table 3). Due to discharge from the NICU which led to discontinuation of study participation, it is likely there were too few subjects included at this time point to detect a statistically significant difference in 25(OH)D levels between the two groups. After 8 weeks of treatment; however, a larger proportion of patients receiving the 800-dose level had 25(OH)D_3_ levels above 75 nmol/l relative to patients receiving the 400-dose level (89% vs. 73%, p = 0.59) however, this did not reach statistical significance with no reportable differences in the two groups at this time point. Infant 25(OH)D levels had significant change between week 4 and 8 (p = 0.0003), 400 group (p = 0.0186) and 800 group (p = 0.0156) indicating that neither group had achieved steady state during the NICU hospitalization.

**Table 3:** Response to supplementation 400 IU vs. 800 IU of vitamin D_3_.

Variable	400 IU			800 IU			P-value
	N	Median	IQR	N	Median	IQR	
**Cord 25(OH)D nmol/L**	14	41.9	23.5	12	42.9	40.7	0.86
**4 week 25(OH)D nmol/L**	16	84.6	42.9	16	105.3	46.9	0.048
**8 week 25(OH)D nmol/L**	11	114.1	145.5	8	138.78	103.3	0.3
**4 week PTH pg/ml**	16	39	27	16	41	23.5	0.82
**8 Week PTH pg/ml**	12	32.5	28.5	10	40	23	0.49
**4 week Ca**^**++**^ **mg/dL**	16	9.9	0.6	16	10.15	0.5	0.33
**8 week Ca**^**++**^ **mg/dL**	12	10.3	0.3	10	10.15	0.5	0.22
**Change in 25(OH)D Cord to 4 week, nmol/L**	14	45.7	38.4	12	71.9	62.9	0.27
**Change in 25(OH)D Cord to 8 week, nmol/L**	9	65.1	39.1	6	72.6	122.3	0.38
**Length of Stay (days)**	16	52	21	16	59	58.5	0.44
**TPN exposure (weeks)**	16	1	1	16	1	1	0.45

### Growth/Adverse events

Median growth chart (Fenton 2003) percentile ranking for length at 36 weeks CGA in the group receiving 800 IU/day compared to the group receiving 400 IU/day trended toward significance (28^th^ vs.19^th^ percentile, p = 0.06), and the 800 IU/day group showed a non-significant (likely due to reduced sample size) higher percentile ranking for weight at 36 weeks CGA (28^th^ vs. 18^th^ percentile respectively, p = 0.14), again without a statistical difference. There were no significant differences in adverse outcomes between the two groups for hyperbilirubenimia requiring phototherapy (15 in each dose group p = 1), need for chronic diuretics (2 in each dose group, p = 1), or chronic lung disease (400 IU group 6, 800 IU 3, p = 0.43).

### Calcium metabolism and DEXA scan

**Fig 2:**
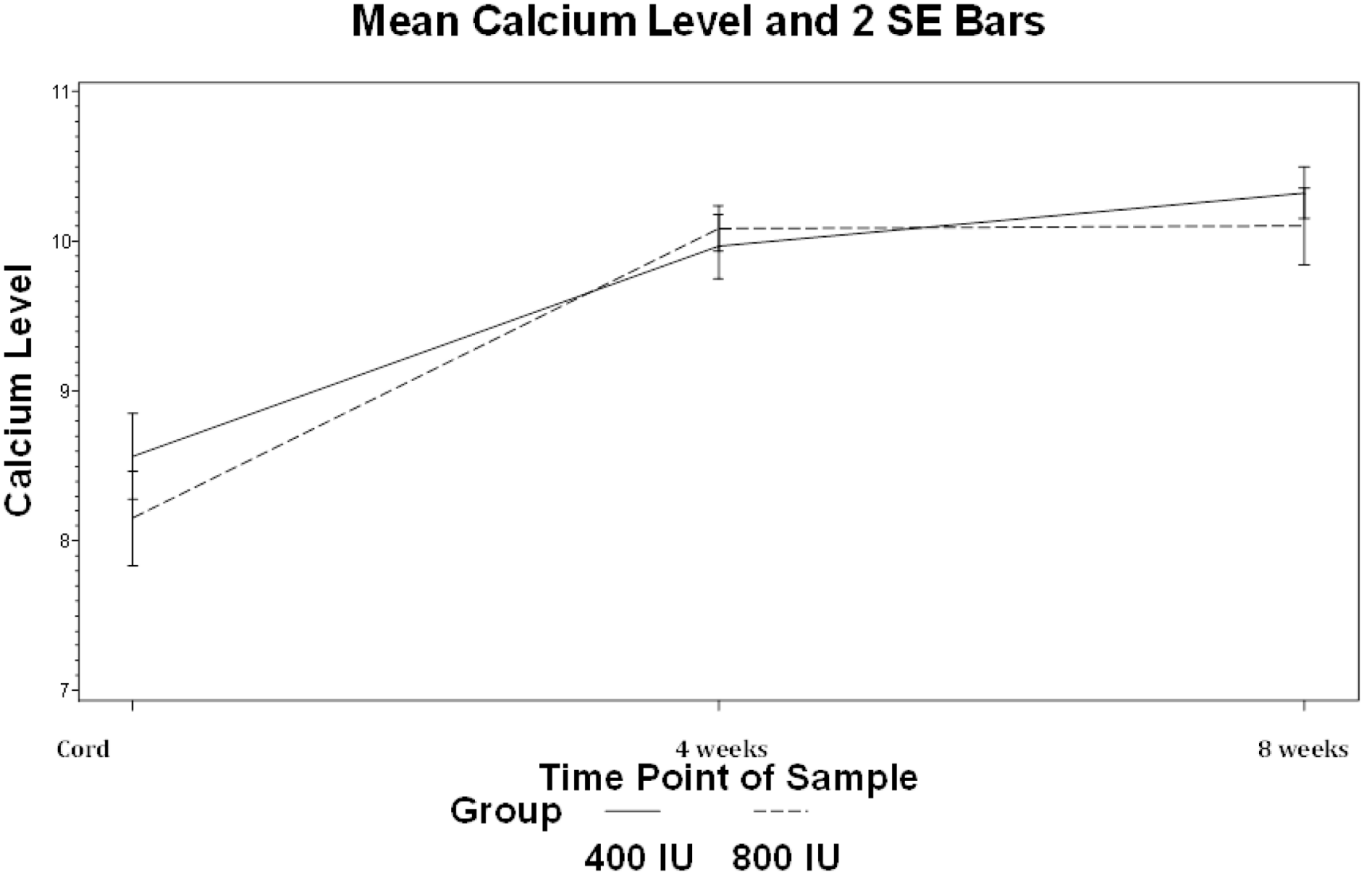
Mean calcium levels and 2 SD bars. Mean calcium (mg/dL) levels were not different at any time point during supplementation with 400 or 800 IU of vitamin D.

There was no difference in serum calcium levels or hypercalcemia at any point between the two groups (Fig 2). While there was no difference in bone mineral density (BMD) between the two groups, a higher proportion of infants in the 400 IU group had global DEXA bone mineral content (BMC) measurements <10 percentile (56% vs 16% p = 0.04) [34]. DEXA Fat and Fat Free Mass was not different between the two groups (p = 0.44) Mean corrected gestational age at DEXA was 39.5 weeks and was not different between the two groups.

### Dietary vitamin D intake

Vitamin D intake from diet was calculated for subjects by week of study. There were statistically but not clinically significant differences in the intake of vitamin D from diet favoring the 400 IU group during the 1^st^ and 7^th^ weeks of the study (Table 4). No significant trends were associated with season of birth or with PTH level (measured at 4 and 8 weeks) at any study time point.

**Table 4:** Weekly dietary vitamin D Intake by group.

Vitamin D Intake IU/day	400 IU			800 IU			p-value
	N	Median	IQR	N	Median	IQR	
**Week 1**	16	233	116	16	126	117	0.046
**Week 2**	16	267	109	16	289	196	0.75
**Week 3**	16	327	154	16	351	142	0.99
**Week 4**	16	357	186	16	329	190	0.64
**Week 5**	15	399	244	14	313	266	0.44
**Week 6**	14	455	174	10	245	220	0.11
**Week 7**	11	412	291	9	238	95	0.04
**Week 8**	9	380	350	7	339	134	0.17

### Results for infants <1200 grams at birth

As some of the smallest and youngest patients to be enrolled in a vitamin D trial, infants in the study <1200 grams at birth were evaluated for 25(OH)D_3_ status and response to supplementation regardless of supplementation group, and compared to the larger infants in the study. These results are shown in Table 5.

**Table 5:** Characteristics of infants <1200 grams as compared to the remainder of the cohort.

Variable	Birth Weight <1200 grams			Birth Weight > = 1200 grams		
	N	Median	IQR	N	Median	IQR
**CGA at Birth weeks**	8	26.65	4.48	24	31.35	1.7
**Birth Weight grams**	8	900	387	24	1513	282
**Birth Weight %ile**	8	35.5	18	24	37	41.5
**Birth Length cm**	8	32.9	3.5	24	40.75	3.5
**Birth Length %ile**	8	11.5	13	24	24.5	50.5
**Birth HC cm**	8	24.75	4.15	24	28.2	2.25
**Birth HC %ile**	8	16	34	24	36	41
**Cord 25(OH)D nmol/l**	4	32.45	22.67	22	45.8	31.4
**4 week 25(OH)D nmol/l**	8	95.77	27.6	24	88.96	63.7
**8 week 25(OH)D nmol/l**	7	148.11	126	12	117.59	105.7
**4 week PTH pg/ml**	8	41.5	46.5	24	38.5	22.5
**8 Week PTH pg/ml**	7	35	11	15	37	33
**4 week Ca**^**++**^ **mg/dL**	8	10.05	0.85	24	10.05	0.55
**8 week Ca**^**++**^ **mg/dL**	7	10.4	0.7	15	10.2	0.3
**Change in 25(OH)D Cord to 4 week nmol/l**	4	51.84	44.9	22	46.6	52.4
**Change in 25(OH)D Cord to 8 week nmol/l**	4	84.26	122.3	11	66.69	127.3

## Discussion

Our study demonstrated a significant improvement in 25(OH)D_3_ status of premature infants with vitamin D_3_ supplementation, with a higher proportion of infants in the 800 IU group achieving desired serum 25(OH)D_3_ concentrations above 75 nmol/l at 4 weeks. Our group of preterm infants at birth had suboptimal vitamin D status with the median of both groups demonstrating levels below 75 nmol/l. Preterm infants fed breast milk or formula without supplementation have been shown in previous studies to have decreasing levels over the subsequent weeks (28%) or to maintain their 25(OH)D status at suboptimal levels [32]. We showed significant improvements in vitamin D status between the groups supplemented with 400 IU and 800 IU at four weeks of age. As the number of participants in each group decreased over time, we believe this warrants further investigation. After eight weeks of supplementation, the group of infants supplemented with 400 IU of vitamin D_3_ had 27% of infants with levels of 25(OH)D_3_ less than <75 nmol/l as compared to only 11% of infants with 25(OH)D <75 nmol/l in the group supplemented with 800 IU D_3_. (p = 0.59) Natarajan et al reported improvement in 25(OH)D levels in preterm infants 28–34 weeks when using a target of >50 nmol/l in a group supplemented with 800 IU as compared to 400 IU. 67% of this cohort supplemented with 400 IU daily had levels <50 nmol/l at the completion of the study period [35]. A notable difference between this study and ours is that in Natarajan’s intervention the dose of vitamin D supplementation was reduced to account for dietary intake of vitamin D and the total intake of vitamin D was 400 IU or 800 IU. In our study supplementation of vitamin D was in addition to dietary vitamin D leading to greater total daily intake. In our study, several infants in the 400 IU group had levels lower than cord levels at both 4 and 8 weeks, but no infants in the 800 IU group had a decrease in 25(OH)D levels, evidence that 400 IU of supplementation in this high-risk group may not be adequate.

The AAP and the IOM support maintaining 25(OH)D levels >50 nmol/l in order to promote optimal bone health [5,6,8]. While both study populations did achieve this lower median target with supplementation at either 400 or 800 IU of vitamin D_3_ by the fourth week of study intervention, a number of individual subjects in both groups did not achieve this target by four weeks (n = 4) of study intervention. This highlights our concern that while the described intervention at either dose is successful for achieving mean targets >75 nmol/l in both groups in composite, individual subjects remain at risk for bone hypo-mineralization (rickets or bone disease of prematurity) despite standard (400 IU) or higher dose (800 IU) intervention.

Bone health has long been the primary association with vitamin D in infants and children. Initial studies of and recommendations for vitamin D intake were designed to prevent rickets in term infants targeting a serum 25(OH)D level of >50 nmol/l [36,37]. The preterm population of patients is at high risk for bone disease of prematurity, and improving delivery of vitamin D may have a positive impact on bone health. Improving vitamin D alone is not adequate to prevent poor bone mineralization; this physiology is also dependent on delivery of appropriate amounts and ratios of calcium and phosphorus in the parenteral and enteral diet, as well as a patient’s over all nutrition status [21]. Elevation of alkaline phosphatase has historically been associated with bone disease of prematurity however in our practice we have found that this marker normalizes in VLBW populations given improved calcium and phosphorus from birth and therefore lacks sensitivity to more subtle variations in bone health status in this patient population. No significant difference was appreciated in PTH at either 4 or 8 weeks between the two dosing groups. This may be due to lack of maturity of the PTH axis in infants of this gestational age or reflective of the overall 25(OH)D status of each group with small numbers not being powered to reach significance.

Evaluation of bone density by DEXA scan is one of several methods available to evaluate bone health in premature infants. Rigo et al have described detailed bone density curves with DEXA for preterm infants allowing for standardization of evaluation in this population [34]. In other measures of preterm infant growth, measurements below the 10^th^ percentile indicate intra- or extra-uterine growth failure [38,39]. Using this 10^th^ percentile standard, a significantly higher proportion of infants in the 400 IU vitamin D group were at risk for low bone density than their peers in the 800 IU dosing group (56% vs 16% p = 0.04 n = 16). Other methods of evaluating bone health include ultrasound and chemical markers in serum and urine such as alkaline phosphatase or osteocalcin and deoxypyridinoline, which were outside the scope of this study [40,41]. Of note, there is no gold standard of evaluation in this challenging population [40,42].

No patients developed hypervitaminosis D as defined by tetany, hypercalcemia, or clinical intolerance of study drug on either dose, even with the wide variation in response to supplementation in the two populations with a maximum increase in 25(OH)D level of 252.7 nmol/l in the 800 IU D3 group [38], and a minimum change in 25(OH)D level of -2.87 nmol/l in the 400 IU group. It is likely that for a proportion of high-risk preterm infants a dose of 400 IU is adequate for normal cellular and tissue function, however there is a significant proportion of the population which does not adequately respond to this dose and in which a higher dose such as 800 IU or 1200 IU may be advantageous. As reported by Gallo et al, a population of healthy breastfed infants required up to 1600 IU daily to achieve increased plasma 25(OH)D concentrations of >75 nmol/L [43]. No studies using doses from 400–1600 IU daily in this patient population have reported undesired side effects [29,35,43–45].

The smallest infants in our study had responses to vitamin D_3_ supplementation similar to their larger peers despite dosing in total IU per day instead of a weight based dosing. This response without evidence of overdose at either dosing level in the smallest infants helps to support the safety and efficacy of these recommended doses for infants less than 1200 grams, a question that has been raised by others [6].

After 8 weeks of supplementation of preterm infants with 800 IU, 11% of infants have 25(OH)D levels below 75 nmol/l, the level that many including the Endocrine Society consider optimal for both skeletal and extra-skeletal effects [9,46]. This, coupled with the lack of dangerously high levels or clinical side effects reported with dosing between 400–1600 IU in preterm infants, lead us to argue for consideration of and additional evaluation of the 800 IU dose of vitamin D in addition to vitamin D from diet sources in this high-risk hospitalized patient population. Reports of clinical effects from vitamin D overdose in infants and children are quite rare. In a case series of 7 children aged 0.7–4.2 years, Kara et al report intake between 66,000 and 800,000 IU daily for periods of up to two months in an erroneously produced fish oil product containing 800,000 IU of vitamin D/dose. Serum 25(OH)D mean levels were 1547.5 nmol/l and patients presented with hypercalcemia, vomiting, anorexia, fever, weakness and constipation. All patients were hospitalized for IV therapy but no lasting complications were documented [47]. Jones has outlined a mechanism by which this toxicity may present arguing that 25(OH)D_3_ at these concentrations displaces 1-alpha,25(OH)_2_D from vitamin D binding protein leading to increased gene transcription [48]. Given serum response to such high doses over a 2-month period, it is unlikely that response would be toxic or lead to hypervitaminosis D when dosing at 800 IU/day throughout the course of NICU hospitalization.

One group of neonates would be at risk from such dosing recommendations that is infants with Williams syndrome, formerly known as idiopathic hypercalcemia of infancy, a genetic syndrome associated with a deletion of 27 genes on chromosome 7 [49]. These patients are often dysmorphic with a wide and slack mouth, underdeveloped mandible, elfin facies, supravalvular aortic stenosis, low set ears, craniostenosis, and increased bone density [50]. Patients will have hypercalcemia and some will show abnormal accumulation of 25(OH)D, however most will have normal 25(OH)D_2_ and 25(OH)D_3_ levels while hypercalcemic or normocalcemic [50]. Supplementation of these patients with vitamin D would not be advisable. Hospitalized preterm infants will have early and often frequent evaluation of calcium levels in the course of their normal care, so inadvertent hypercalcemia is unlikely to go undiagnosed in a <32-week preterm patient population.

Trends toward improved growth in both length and weight are also important outcomes that require additional exploration. Growth in the NICU period has a direct correlation to neurodevelopmental outcomes, and is critical to an infant’s long-term success [51]. Additional research is necessary, particularly for smaller and more preterm infants who are at high risk for development of bone demineralization, rickets of prematurity, extrauterine growth failure, and bronchopulmonary dysplasia, all of which have the potential to be positively impacted by improved 25(OH)D levels.

This study is the only study to date to evaluate infants as small as 24 weeks EGA; others have reported data on infants 28 weeks and greater. These infants are the highest risk infants and are in need of additional evaluation of vitamin D status and response to supplementation. Our DEXA scan evaluation of these infants at term corrected age is an important indicator of both short term and long-term benefits of improvement in vitamin D status. Our study, however; does have limitations. We were only able to evaluate 32 infants, which limits the statistical significance of some of our outcomes. Additionally, a number of infants (16) did not complete the outpatient DEXA scan despite multiple attempts to schedule and reschedule the scan and to accommodate any special travel or transportation needs of the family. We were unable to adequately assess maternal vitamin D intake during pregnancy in the cohort studied, as maternal records often did not report the amount of vitamin D in the prescribed prenatal vitamin. However, given that the majority of prenatal vitamins have 400 IU of vitamin D and this has not been shown to increase levels in pregnancy this likely does not have any impact on our described results.

## Conclusions

Administration of 400 or 800 IU of vitamin D_3_ improves vitamin D status at 4 weeks as compared to cord levers in high risk infants <32 weeks EGA at delivery. Dosing vitamin D_3_ daily at 800 IU in this population significantly improves 4 week 25(OH)D_3_ status as compared to a 400 IU daily dose. Additionally, risk of bone density less than the 10^th^ percentile as measured by DEXA at term corrected age is decreased with a dose of 800 IU daily while in the NICU. Although this study was underpowered to show a statistical difference between the two dosing levels at all points of evaluation, not all infants achieved levels of 75 nmol/L at 4 or 8-week measurements, and a number of infants have levels <50 nmol/l at 4 weeks. There was also a trend towards improvement in linear growth in the infants dosed at 800IU of vitamin D_3_ daily as compared to the lower dose. Our results support a daily supplementation dose of 800 IU as compared to 400 IU in this at risk extremely preterm patient population. Evidence continues to mount in support of this supplementation of preterm infants with vitamin D early in hospitalization [44]. Vitamin D toxicity is a rare event, and tolerance of even higher doses of vitamin D have been reported in a variety of populations [9,52]. Alternatively, the risks of recommendation of too low a dose may have significant impact on individual and population health with disease outcomes associated with low vitamin D status, including hypocalcemic seizures, growth restriction, fractures, and elevation of serum parathyroid hormone [53–55]. In fact, some authors, including Heaney, have argued that a physiological reference for optimal vitamin D status would be between 100 and 130 nmol/L stating that based on physiological outcomes such as normalization of PTH and adequate vitamin D supply in breast milk this level is safe for disease prevention without association of toxicity [56]. Other authors have recommended loading doses to more quickly improve vitamin D status in at risk populations, a strategy that merits further investigation [57].

Our results agree with previous evidence that supports an association between vitamin D status, gestational age, and growth in premature infants. The optimal dose of vitamin D supplementation for preterm infants during the early neonatal period warrants further investigation, but it is reasonable and safe to recommend daily supplementation in addition to dietary intake with 800 IU of vitamin D to high-risk preterm infants while hospitalized in the NICU in order to target a level of 25(OH)D that is >75 nmol/l, as we have shown this to improve bone health defined by DEXA in this at-risk population.

## Supporting information

S1 File CONSORT checklist. (DOC)

S2 File Trial protocol. (PDF)

S3 File Relevant data. (XLSX)
