# Supplementary material for: Randomized trial of two doses of vitamin D3 in preterm infants <32 weeks: Dose impact on achieving desired serum 25(OH)D3 in a NICU population
Source: PLoS One. 2017 Oct 10;12(10):e0185950. doi: 10.1371/journal.pone.0185950 (PMC5634602; doi:10.1371/journal.pone.0185950)
Supplement: S2 File — (PDF) [file pone.0185950.s002.pdf]

Medical Center

PEDIATRIC BIOMEDICAL RESEARCH APPLICATION

SECTION I

1. STATUS:

☐ **New Submission**
☒ **Revised protocol, IRB #** 419-11 FB**; version # or date** v3, 3/1/2012

2. TITLE OF PROTOCOL:

SERUM 25(OH)D LEVELS, SUPPLEMETAL VITAMIN D, AND PARATHYROID HORMONE LEVELS IN PREMATURE INFANTS

3. RESPONSIBLE PERSONNEL

Do not use nicknames. **In order to verify CITI training, list each individual's full name (i.e., first, middle and last) and degree. The name should match the legal name utilized by University of Nebraska Medical Center/The Nebraska Medical Center, University of Nebraska – Omaha or Children's Hospital and Medical Center (UNMC, TNMC, UNO or CHMC).**

A. PRINCIPAL INVESTIGATOR (PI)

**Name:**	Corrine Hanson, PhD, RD	**Position: Assistant Professor**	
**Department:**	Medical Nutrition Education	**Campus Zip:4045**	
**Address:**	4045 Nebraska Medical Center		
**Email address:**	ckhanson@unmc.edu		
**Phone:**	402-559-3658	**Pager:**	

*Note: This individual assumes **overall** responsibility for 1) development and submission of this IRB application, 2) obtainment of legally effective informed consent and assent (as applicable) from prospective subjects by all authorized personnel listed on this IRB application, 3) the performance of research interventions, 4) conduct of the research in full compliance with the Common Rule, applicable Subparts B, C, and D of HHS regulations at 45 CFR 46, applicable FDA regulations, the HIPAA Rule, applicable state law, HRPP policies, the provisions of the protocol as approved by the IRB, and 5) the presentation or publication of the data. Only **one** PI can be named on the IRB application. Co-PIs (e.g., on NIH grants) must be listed as Secondary Investigators.*

If the PI is a student (e.g., medical, dental, pharmacy, nursing, allied health, undergraduate, graduate), a faculty advisor must be listed and must sign the Faculty Certification in Section I.10. The faculty advisor assumes responsibility for overall supervision of the student's research.

B. SECONDARY INVESTIGATOR (SI)

Names	Position	Department
Ann Anderson Berry, MD	Associate Professor	Pediatrics
Laura Armas, MD	Assistant Professor	Creighton Osteoporosis

		Research Center
John Wendell		Childrens Hospital and Medical Center

Note: The SI(s) may also be termed co-investigators and share responsibility with the PI for: 1) development and submission of the application to the IRB, 2) obtainment of legally effective informed consent/assent from prospective subjects (as applicable), 3) performance of research interventions, 4) conduct of the research in full compliance with the Common Rule, applicable Subparts B, C, and D of HHS regulations at 45 CFR 46, applicable FDA regulations, the HIPAA Rule, applicable state law, HRPP policies, the provisions of the protocol as approved by the IRB, and 5) the presentation or publication of the data.

C. PARTICIPATING PHYSICIANS/PERSONNEL

Names	Position	Department
Melissa Thoene	Medical Nutrition Therapist	Pharmacy and Nutrition Care
Laura Dugick	NNP	NICU
Julie Wagner	Student	Medical Nutrition Education

Note: These individuals are not involved in the development and submission of the application to the IRB, but may be involved with taking care of patients on protocol as part of their normal clinical duties, and obtainment of legally effective informed consent/assent from prospective subjects. All participating personnel must have sufficient knowledge about the protocol to facilitate effective interaction with the subject in a clinical context.

D. LEAD COORDINATOR

Name	Phone	Pager	Email

*Note: This individual serves as the primary contact point for the Office of Regulatory Affairs (ORA) and is copied on all email notifications generated by the ORA. This individual may be administratively involved in IRB submissions and may also be involved in conducting study-related procedures, obtainment of legally effective informed consent/assent in accordance with HRPP policies, and taking care of the subject. Only **one** Lead Coordinator can be named. Other staff providing back-up for the Lead Coordinator should be classified as "Coordinator".*

E. COORDINATOR

Names	Phone	Email

Note: This individual(s) serve as a secondary contact point for the ORA and is copied on all email notifications generated by the ORA. This individual(s) may be administratively involved in IRB submissions and/or may serve as regulatory staff for other review committees. This individual(s) may also be involved in conducting study-related procedures and obtaining legally effective informed consent/assent in accordance with HRPP policies.

F. DATA AND ADMINISTRATIVE PERSONNEL:

Names	Phone	Email
Elizabeth Lyden	402-559-6061	elyden@unmc.edu
Jeff Larson	402-559-7422	jlarson@unmc.edu

Note: These individuals do not have direct contact with subjects, but may have access to subject's identifiable protected health information (PHI) and may be directly involved in data management, research budget management (including research billing), and statistical support. These individuals usually do not serve as a contact for the ORA.

4. FUNDING SOURCE: Check all that apply and provide the source of the funding. *Note: All research must have a source of funding. Examples of other funding sources may be departmental funds, Clinical Research Center Support grants, or personal funding.*

☒ Grant – Provide source:
Edna Ittner Foundation

☐ Commercial – Provide company name:

☐ Other – Provide source:

☒ Center for Clinical and Translational Research (CCTR)

5. CONTRACT: Is there a contract or agreement associated with this study? ☐ Yes ☒ No

6. FUNDING AGENCY DEADLINE FOR IRB APPROVAL:

7. STUDY SITES:

A. Provide the names and locations of all study sites where this research will be conducted under the oversight of the PedsIRB. *Note: Study sites are defined as all internal or external locations, such as a clinic, laboratory, school, center where UNMC, TNMC, UNO or CHMC investigators or staff interact with subjects, collect data or solicit consent (e.g., UNMC, TNMC, UNO, CHMC, Turner Park Clinic, Monroe Meyer Institute, individual physician offices, Millard North High School.)*

**UNMC**	
CHMC	

B. If this is a multi-institutional protocol where UNMC, TNMC, UNO or CHMC serves as the lead site with responsibility for data and/or safety monitoring, provide a list of all sites where this study will be conducted. *Note: This section should specify study sites at institutions that are under the oversight of external IRBs (e.g., Omaha VA Medical Center, other academic health science centers or hospitals, other universities).*

8. PRINCIPAL INVESTIGATOR ASSURANCE

The PI understands and accepts the following obligations to protect the rights and welfare of research subjects in this study:

- I certify that I, and all listed research personnel, have the necessary qualifications, expertise, and hospital credentials to conduct this study in a manner which fully protects the rights and welfare of research subjects.
- I certify that all listed Secondary Investigators have been given a copy of this IRB application and any other relevant study related documents and have agreed to be a Secondary Investigator.
- I certify that all listed Secondary Investigators and other involved research personnel will be given a copy of the final IRB approved application and any other relevant study related documents.
- I recognize that, as the PI, it is my responsibility to ensure that this research and the actions of all research personnel involved in conducting the study will comply fully with the IRB-approved protocol, all applicable federal regulations, state laws, and HRPP policies.
- I recognize that it is my responsibility to ensure that valid informed consent/assent has been obtained, as appropriate, from all research subjects or their legally authorized representative (LARs). I will ensure that all research personnel involved in the process of consent/assent are properly trained and are fully aware of their responsibilities relative to the obtainment of informed consent/assent according to HRPP policies, applicable federal regulations, and state law.
- I certify that the minimum amount of protected health information (PHI) necessary will be used and disclosed to conduct this research study (if applicable). I will implement reasonable safeguards to protect the PHI at all times.
- I will promptly inform the IRB of internal adverse events, as well as any unanticipated problems involving risk to the subjects or to others, as required within the time frame defined by HRPP policies.
- I will analyze each MedWatch/safety report to determine if it impacts the risk benefit relationship of the study, the safety of the subjects, or informed consent. I will promptly submit external adverse event reports in accordance with IRB requirements.
- I will promptly inform the IRB if I become aware of: 1) any complaints from research subjects, LARs, or others about research participation, 2) violations of federal regulations or state law, 3) violations of the HIPAA Rule, or 4) violations of HRPP policies.
- I will promptly inform the IRB of the results of external audits performed by sponsors, Contract Review Organizations (CROs), cooperative groups, FDA, or other external groups.
- I will not initiate any change in protocol without IRB approval except when it is necessary to reduce or eliminate a risk to the subject, in which case the IRB will be notified as soon as possible.
- I certify that there are, or will be, adequate resources and facilities to safely initiate, carry out and complete this research at the study sites specified in Section I.9. This includes sufficient staff, funding, space, record keeping capability, and resources necessary to address adverse events and any unanticipated problems involving risk to the subject or others. If the necessary resources become unavailable I will promptly notify the IRB.
- I will promptly inform the IRB of any significant negative change in the risk/benefit relationship of the research as originally presented in the protocol and approved by the IRB.

- I understand that continuing review by the IRB is required at least annually in order to maintain approval status. I will maintain IRB approval as long as this study is active.
- I understand that I am responsible for appropriate research billing in accordance with *UNMC Clinical Trial Professional and Technical Fee Billing Policy #8080* or applicable CHMC policy.
- I will maintain all required research records on file and I recognize that representatives from the IRB, OHRP, HHS, FDA, and other Federal Departments or Agencies may inspect these records in accordance with granted authority.
- I certify that I and all other personnel listed in Section I of the IRB Application have disclosed all potential financial conflicts of interest as required and are in full compliance with the UNMC Conflict of Interest Policy. I further certify that all potential financial conflicts of interest are appropriately managed in order to ensure protection of the rights and welfare of patients.
- I understand that failure to comply with the Common Rule, applicable Subparts B, C, and D of HHS regulations at 45 CFR 46, applicable FDA regulations, the HIPAA Rule, applicable state law, HRPP policies, and the provisions of the IRB-approved protocol may result in suspension or termination of IRB Approval of my research project and/or other administrative or legal actions.

____
Printed Name of Principal Investigator

______________________________ ____________
Signature of Principal Investigator Date

9. CERTIFICATION OF FACULTY ADVISOR

If the PI is a student (e.g., medical, dental, pharmacy, nursing, allied health, undergraduate, graduate):

My signature certifies that I have reviewed this IRB application and approve it for submission to the IRB. I assume responsibility for the overall supervision of this research. I will advise the student on the responsible conduct of research including compliance with all applicable federal regulations, state laws, and HRPP policies.

____
Printed Name of Faculty Advisor

______________________________ ____________
Signature of Faculty Advisor Date

10. PRINCIPAL INVESTIGATOR FINANCIAL INTEREST DISCLOSURE

A. ***As the PI, I certify that I am in full compliance with UNMC Conflict of Interest Policy 8010 and I declare:***

1) ☒ I have no financial interest in this research.

OR

☐ I have a financial interest in this research. I have completed the *UNMC Disclosure of Potential Conflict of Interest Form* and obtained all required signatures. The original disclosure form is attached to this application. *Note: A COI management plan for the PI conflict of interest must be developed before this application will be reviewed by the IRB.*

2) ☐ I understand that if there is any change in my financial interest during the course of this research, I will update and submit the *UNMC Disclosure of Potential Conflict of Interest Form* within five (5) business days from the time the change becomes known.

B. ***As the PI who is ultimately responsible for the proper conduct of this research, I also certify that:***

1) ☒ No Responsible Personnel have a financial interest in this research.

OR

☐ The Responsible Personnel listed below have informed me that they have a financial interest in this research. Each person identified below has completed the *UNMC Disclosure of Potential Conflict of Interest Form* and obtained all required signatures. The original disclosure form is attached to this application.

______________________________ ______________________________

______________________________ ______________________________

2) ☐ I have informed all Responsible Personnel that if there is any change in their financial interests during the course of this study it must be disclosed by submitting or updating the required *UNMC Disclosure of Potential Conflict of Interest Form*.

______________________________ ____________

Signature of Principal Investigator Date

In accordance with 1) UNMC Conflict of Interest Policy 8010, and 2) HRPP Policy 1.11, ***all*** *actual or potential financial conflicts of interest of Responsible Personnel ("covered persons") must be identified and minimized through appropriate management. Responsible Personnel are defined as the Principal Investigator, Secondary Investigator, Participating Personnel, Lead Coordinator, and Coordinator. Covered persons also includes a Responsible Person's immediate family (i.e., parent(s), or spouse of a parent, spouse, dependent child of a covered person, or anyone that a Covered Person may claim as a dependent under the Internal Revenue Code will be treated as a financial interest of the Covered Person.)*

Financial interest may include, but is not limited to, any current or potential:

- *Consulting relationship including speakers bureaus, advisory board member or other work on behalf of the sponsor outside of university grants/contracts*
- *Ownership of any intellectual property (patent, copyright or royalty)*
- *Equity interest (stocks/other company ownership, except for mutual funds)*
- *Personal loans*
- *Director, officer, employee or owner*
- *Other payment or financial remuneration involving you or your immediate family and a sponsor*.

11. SCIENTIFIC/SCHOLARLY MERIT AND RESOURCE REVIEW CERTIFICATION

Note: Research proposals must undergo substantive scientific/scholarly merit and resource review ***prior to submission*** *of the application to the IRB. This IRB Application must provide evidence of this review. IRB applications submitted without this certification will* ***not*** *be reviewed by the IRB.*

The chairperson, authorized delegate, or appointed review committee of the PI's school, department or division is responsible for review of the research proposal ***prior*** *to submission. A Chairperson/delegate who is also listed as study personnel in Section I of this application cannot provide certification.*

My signature certifies that this application has been reviewed for scientific/scholarly merit and available resources. I have determined that the application merits consideration by the IRB based upon the following:

1) The proposal has an acceptable level of scientific/scholarly merit which justifies the use of human subjects.

2) The proposal has a sound research design which will achieve the stated objectives,

3) The PI has the necessary qualifications and experience to conduct this research.

4) The PI has or will have the necessary funding to support this research.

5) There is adequate physical space required for the research interventions at all study sites specified in Section 1.8. In addition, there is adequate laboratory and clerical support, data storage capability, and any other resources necessary to complete this research.

6) At all study sites specified in Section 1.8, there is emergency equipment, personnel, or services necessary to respond promptly to adverse events or unanticipated problems involving risk to the subject or others.

7) I will promptly notify the IRB if the necessary resources to support this research become unavailable.

I am not listed as study personnel in Section I of this application.

______________________________	______________________________
Printed Name of Reviewer	Position
______________________________	__________
Signature of Reviewer	Date

SECTION II

Instructions: In order to review your proposal, the IRB must have the following information pursuant to its charge by federal regulations for the protection of human subjects. Each subpart must be titled using numbered **boldface subheadings** as described below and addressed independently in the listed sequence without reliance on information covered under other subparts. Attachment of applicable sections of the grant application or detailed protocol is not acceptable as a substitute for completion of each subpart.

*The IRB Application must provide sufficient information to facilitate an effective review by all members of the IRB including non-specialists. There is an educational guide to this application available on the IRB website titled "**Educational Guide – Biomedical Research (PedsIRB)**". Questions that have an educational note that explain the information requirements will be referred to the Guide with the statement "See Educational Guide – Biomedical Research (PedsIRB)". Applications that do not allow for an effective review may be returned to the investigator, without IRB review, for revision and resubmission.*

PROTOCOL ABSTRACT

Provide a brief (less than 400 words) abstract of the research protocol. This summary should include: 1) the title of the protocol, 2) a *brief* description of the purpose of the study, 3) eligibility criteria, 4) interventions and evaluations and 4) follow-up.

Serum 25(OH)D levels, Supplemental Vitamin D, and Parathyroid Hormone Levels in Newborn Hospitalized Infants

Low maternal serum 25(OH)D levels have documented negative repercussions on the skeletal health of the newborn. Parathyroid hormone (PTH) levels are often used as a marker of vitamin D sufficiency, however 25(OH)D levels to achieve suppression of PTH levels in premature infants is unknown. In addition, recent evidence has shown that vitamin D insufficiency (defined as 25(OH)D levels of <30 ng/mL) is an important risk factor in for neonates and children acquiring infections such as RTI and pneumonia.

The specific aims of this study are to determine levels of vitamin D supplementation to achieve goal serum 25(OH)D levels of 30 ng/mL, and to define serum 25(OH)D levels required to achieve suppression of parathyroid hormone in preterm newborn infants hospitalized in the Nebraska Medical Center Newborn Intensive Care Nursery (NICU). In this randomized, controlled trial, infants 23 weeks gestational age or greater will be randomized to two different levels of vitamin D supplementation: 400 IU vitamin D3/day, or 800 IU vitamin D3/day. As an exploratory aim, vitamin D binding protein levels (VDBP) and term corrected age DEXA scan to evaluate bone mineralization will also be quantified in these infants.

This research is innovative because while vitamin D research is occurring in many arenas, few investigators have ethical access to a population of hospitalized infants, including infants born prematurely. Bone health is a special concern in this population. A high percentage of bone mineralization takes place in the last trimester of pregnancy, and premature infants should be rapidly mineralizing bone. While a high prevalence of vitamin D deficiency is being documented in this population, less is known about vitamin D's relationship with PTH. We seek to define intakes of vitamin D that will result in goal

25(OH)D levels, and of serum concentrations of 25(OH)D that will suppress PTH in a population of hospitalized, premature infants.

PURPOSE OF THE STUDY AND THE BACKGROUND (1-2)

1. PURPOSE OF THE STUDY
What are the specific scientific objectives (aims) of the research?

• Primary Specific Aims:

- To investigate a dose of vitamin D supplementation necessary to achieve goal 25(OH)D levels of 30 ng/mL in a population of premature infants during their NICU hospitalization.
- To determine the serum 25(OH)D level required to achieve suppression of parathyroid hormone in a population of premature, hospitalized infants.

• Secondary Specific Aims:

- As a secondary, exploratory aim, vitamin D binding protein and its relationship with 25(OH)D levels will be measured and quantified in a population of premature, hospitalized infants. A term corrected age DEXA scan to evaluate bone mineral content will also be performed.

2. BACKGROUND AND RATIONALE
Describe the background of the study. Include a critical evaluation of existing knowledge, and specifically identify the information gaps that the project is intended to fill. *See Educational Guide – Biomedical Research (PedsIRB)*

Infants are completely dependent on the maternal supply of vitamin D, and an infant will be born with 25(OH)D levels that are approximately 80% of the mother's circulating 25(OH)D levels.(2) With recent evidence that a large percentage of the population of women of child-bearing age, and especially African-American women, are vitamin D deficient or insufficient, newborns are at high risk for vitamin D deficiency.(3-5)

Low serum 25(OH)D levels have documented negative repercussions on the skeletal health of the newborn. Infants of vitamin D deficient mothers show splaying of the distal metaphysic of the femur and the presence of craniotabes in utero and at birth, both of which are consistent with rickets and lower bone mass relative to body weight at birth (6-8). These deficits persist into the first decade of life, with an increase in bone mineral mass at 8 years of age in vitamin D supplemented infants (9), and a decrease in bone mineral content at 9 years of age in children whose mothers were vitamin D deficient (10). Parathyroid hormone (PTH) is often used as a marker of bone turnover, and an inverse relationship between PTH and 25(OH)D levels has been documented in many studies (11-14). Studies in adolescents show that serum 25(OH)D levels of 36 ng/mL are necessary to suppress PTH levels (14). In addition, recent evidence has shown that vitamin D insufficiency (defined as 25(OH)D levels of <30 ng/mL) is an important risk factor in neonates and children acquiring infections such as respiratory tract infection (RTI) and pneumonia (15,16). Cord blood 25(OH)D levels have been shown to impact innate immune responses (17).

Very little is known about the primary carrier protein of vitamin D, vitamin D binding protein (VDBP) in premature infants. A study conducted in the 1980s show some evidence that VDBP may increase with increasing gestational age (18). These investigators will quantify VDSP in this population, and hypothesize VDSP levels will increase with increased vitamin D supplementation.

Preliminary Studies:

A previous study by these investigators examined the vitamin D and PTH status in a population of hospitalized newborns greater than 32 weeks gestational age who received standard formula feedings with and without 400 IU of supplemental vitamin D3. In this study, the mean (±SD) cord blood level was 17.6 ±7.0 ng/mL. A positive association was found between cord blood 25(OH)D and serum calcium levels in the first week of life (r=0.44, p=0.003) and with gestational age (r=0.35, p=0.015). Neither group achieved goal levels of 30 ng/mL at discharge. Discharge 25(OH)D levels showed a statistically significant negative correlation with parathyroid hormone levels (r=-0.35, p=0.02).

A recent study by Gulati et al showed that in infants <28 weeks gestation, 50% have 25(OH)D levels below 30 ng/mL at 36 weeks CGA, in infants 39-32 weeks, 70% have levels less than 30 ng/mL, and 90% of infants 33-36 weeks have 25(OH)D levels below 30 ng/mL at 36 weeks CGA, when supplemented according to current unit policy. (Results received ahead of publication).

CHARACTERISTICS OF THE SUBJECT POPULATION (3-10)

3. ACCRUAL

A. What is the number of subjects that must complete the study and be evaluable in order to achieve the scientific objectives of the research? *See Educational Guide – Biomedical Research (PedsIRB)*

40 NICU infants who are between 23 and 32 weeks gestational age must complete the study.

B. What is the statistical or other justification for the number of subjects needed to complete the study? *See Educational Guide – Biomedical Research (PedsIRB)*

Based on our previous study, NICU infants who received 400 IU/day of Vitamin D are expected to have a mean discharge serum 25(OH)D of 23.1 ng/ml (standard deviation=7.0). Conservatively, the 800 IU dose group is expected to have a mean discharge serum 25(OH)D of 30 ng/ml (SD=7.0) (SD estimated from our previous study data). Assuming a 0.05 level of significance, and a total drop-out rate of 10 percent; 40 NICU infants randomized to the two dose groups will provide 81 percent power to detect a difference between doses.

C. What is the maximum number of subjects that will be consented at all sites under the oversight of the PedsIRB and what is the justification for this number? *See Educational Guide – Biomedical Research (PedsIRB)*

A maximum of 40 infants who are between 23 and 32 weeks gestational age will be consented for the study.

4. GENDER OF THE SUBJECTS

A. Are there any enrollment restrictions based on gender?

☒ **No**
☐ **Yes. Provide justification.** *See Educational Guide – Biomedical Research (PedsIRB)*

B. Are individuals of childbearing potential excluded from participation in this study?
☐ **No**
☒ **Yes. Provide justification.** *See Educational Guide – Biomedical Research (PedsIRB)*
The study is on infants

C. Are pregnant individuals excluded from participation?
☐ **No**
☒ **Yes. Provide justification.** *See Educational Guide – Biomedical Research (PedsIRB)*
The study is on infants

D. Are breast feeding individuals excluded from participation?
☐ **No**
☒ **Yes. Provide justification.** *See Educational Guide – Biomedical Research (PedsIRB)*
The study is on infants

5. AGE RANGE OF SUBJECTS

A. What is the justification for inclusion of children in this research?

The rationale for the age range included is to add to the current body of knowledge regarding the vitamin D intake required in a population of premature infants to achieve goal 25(OH)D levels and suppression of PTH.

B. What is the age range for the child subjects, and what is the justification for selecting this age range? *See Educational Guide – Biomedical Research (PedsIRB)*

The age range of the subjects will be between 23 and 32 weeks gestational age. This age range will expand upon a previous study completed by these investigators that showed a mean 25(OH)D level of 17 ng/mL in infants greater than 32 weeks gestational age, and recent results by other investigators that show a high prevalence of deficiency in this population. Based on those results, and data that supports a high prevalence of vitamin D deficiency in pregnant women, we seek to define intakes that will promote optimal 25(OH)D levels in a wide population of premature infants.

C. Will this study enroll wards of the state?
☒ **No**
☐ **Yes. Complete the *Addendum H - for Research Involving Children Who Are Wards*.**

D. Will adults (19 years of age or older) be included in this research?
☒ **No**
☐ **Yes. Provide the age range and justification for their inclusion for the stated age range and complete and attach *Addendum Y.***

6. RACE AND ETHNICITY

Are there any subject enrollment restrictions based upon race or ethnic origin?
☒ **No**
☐ **Yes. Explain the nature of the restrictions and provide justification.** *See Educational Guide – Biomedical Research (PedsIRB)*

7. **VULNERABLE SUBJECTS**

A. Will any of the following vulnerable populations be included in this research? *See Educational Guide – Biomedical Research (PedsIRB)*

No	Yes	Vulnerable Population	Complete:
☒	☐	Pregnant individuals, fetuses or neonates (non-viable or of uncertain viability)	Addendum B1
☒	☐	Prisoners	Addendum C

B. Will any other vulnerable population be specifically recruited for enrollment in this research

☒ No

☐ Yes.

1. **Who are the vulnerable subjects that will be specifically recruited for enrollment?** *See Educational Guide – Biomedical Research (PedsIRB)*

2. **Describe the additional protections which will be in place.** *See Educational Guide – Biomedical Research (PedsIRB*

8. **INCLUSION CRITERIA**

What are the specific inclusion criteria? *See Educational Guide – Biomedical Research (PedsIRB)*

Infants who are receiving inpatient care in the Newborn Intensive Care Nursery will be considered for enrollment. Prospective subjects who are between 23 and 32 weeks gestational age will be identified on admission to the Nebraska Medical Center Newborn Intensive Care Nursery.

9. **EXCLUSION CRITERIA**

What are the specific exclusion criteria? *See Educational Guide – Biomedical Research (PedsIRB)*

Exclusion criteria will be infants receiving, congenital abnormalities, GI, liver, or kidney disease, inborn errors of metabolism, parathyroid disease, and disorders of calcium metabolism, and infants receiving seizure mediation or steroids. Infants whose parents are less than 19 years of age will also be excluded from the study, to avoid obtaining parenteral consent from a parent who is not legal adult age in the state of Nebraska. Infants who are made wards of the State of Nebraska will also be excluded from the study, per Nebraska State law 390 NAC 11-002.04K which states wards of the state may not participate in medical research unless a specific exception is granted by the state, following an evaluation of the protocol by staff in the Medical and Legal Divisions of the Nebraska Health and Human Services System.

10. **CONTRACEPTION REQUIREMENTS**

Are there any specific contraception requirements?

☒ No

☐ **Yes. Describe the requirements and provide justification.** *See Educational Guide – Biomedical Research (PedsIRB)*

METHODS AND PROCEDURES (11-15)

11. **METHODS AND PROCEDURES APPLIED TO HUMAN SUBJECTS**

A. Does this protocol require tests to be performed for the purpose of determining subject eligibility which would not be *routinely* conducted as part of standard clinical care of the prospective subject?

☒ **No**

☐ **Yes. Describe the tests to determine subject eligibility.** *See Educational Guide – Biomedical Research (PedsIRB)*

B. Indicate the study design by checking the appropriate boxes.

See Educational Guide – Biomedical Research (PedsIRB)

☐ **Phase I**	☐ **Case-Control**	☐ **Single Blind**
☐ **Phase I/II**	☐ **Cohort**	☒ **Double Blind**
☐ **Phase II**	☐ **Cross-sectional**	☐ **Triple Blind**
☐ **Phase II/III**	☐ **Randomized, parallel group**	
☐ **Phase III**	☐ **Randomized, cross-over**	
☐ **Phase IV**	☐ **Placebo-Controlled**	
☐ **Other:**		

C. Describe sequentially all procedures, interventions and evaluations to be applied to subjects, including follow-up. *See Educational Guide – Biomedical Research (PedsIRB)*

Step 1: Recruitment and Informed Consent

Parents of infants admitted to the NICU who meet eligibility criteria will be approached by study personnel, information about the study will be given, and informed consent will be obtained.

Step 2: Collection of Baseline Data

A sample of cord blood drawn that is drawn at birth for other routine clinical use will be frozen and stored at -70 degrees for study analysis of 25(OH)D levels. Demographic information will be obtained, including gestational age, birth weight, length, circumference, race, ethnicity, and season of birth. This is routine NICU admission information that is not performed exclusively for research purposes.

Step 3: Randomization

Study pharmacist will be notified and eligible infants will be randomized to the 400 IU/day of 800 IU/day supplementation group. Vitamin D supplementation will begins when the infant reaches full enteral feedings. Both the investigators and the subjects will be blinded as to the treatment group.

Step 4: NICU Data Collection

Blood samples will be drawn every 4 weeks, beginning 4 weeks after birth. The maximum amount of blood taken at each blood draw will be 2 mL. The final blood draw will occur at 36 weeks CGA. Analysis of each blood sample will include 25(OH)D levels, PHT levels, and VDBP. A serum calcium will be obtained with each blood draw for safety monitoring purposes, if not already available from routine NICU lab monitoring. Lab draws in the NICU are done by the nursing staff and are done using heelstick or venipuncture, at the discretion of the nursing staff. Nurses may make more than one

attempt to draw blood, but if the lab draw cannot be successfully completed, the attempt is terminated at the discretion of the bedside nurse. Ongoing clinical data collection from the patients' medical record will include daily vitamin D intake, growth parameters, and infections/antibiotic use.

Step 5: Urine collection and DEXA Scan: When the infant reaches 36 weeks gestational age, a 24-hour urine sample will be obtained for measurement of calcium excretion. At 40-44 weeks gestational age, a DEXA scan will be performed.

Test	Frequency of Collection	Amount of Blood
25(OH)D(ng/mL)	Collected on cord blood, every 4 weeks, and at 36 weeks CGA	Requires < 0.5 mL
Serum Calcium (For safety monitoring)	Collected with each 25(OH)D draw	Will be collected for study purposes only when not drawn as part of routine NICU labs (requires 0.5 mL)
**PTH**	Collected with each 2S(OH)D draw	1 mL
**VDBP**	Collected with each 25(OH)D draw	0.1 mL (included in the 0.5 for 2S(OH)D)
**TOTAL**	Max blood draws on any 1 infant, not including cord blood: Every 4 weeks from 24-36weeks: 3	2 mL maximum every 4 weeks A volume of 4 mL in 8 weeks will allow an infant with a weight of 1300 grams to remain within the guidelines of 3 mLlkg of blood for study purposes in and 8 week period. Protocol adjustments will be made to minimize blood volumes in smaller infants

D. Identify any procedures, interventions, and evaluations that are experimental or performed exclusively for research purposes. *See Educational Guide – Biomedical Research (PedsIRB)*

Procedures performed exclusively for research purposes include:
1. Acquisition and storage of cord blood for analysis.
2. Blood samples taken every 4 weeks for analysis of 25(OH)D, iPTH, serum calcium, and VDBP.
3. 24 hour urine collection for calcium excretion
4. DEXA scan after discharge at term corrected age

24-hour urine collection for 24 hour calcium excretion will be collected using an infant urine collection bag that is placed in the infants' diapers. The bags are attached to the skin as per the manufacter's instructions and are changed with every diaper change to minimize any risk of skin breakdown.

A serum calcium levels will be run on the blood sample for study purposes only if the infant does not have a serum calcium level available at the time point (within 72 hours) of the study draw. NICU hospitalized infants have routine clinical monitoring of laboratory values and this routinely includes serum calcium levels. If a serum calcium level is available, it will be used as the value for study purposes, and will decrease the amount of blood required for study purposes.

Guidelines used for blood draw limits for this study was 3 mL/kg over an 8 week period. This volume would allow an infant with a birth weight of 1.3 kg to complete our protocol as written. If the birth weight of the infant is less than 1.3 grams, the volume allowed for the study over the next 8 weeks will be calculated and protocol adjustments will be made. Examples of protocol modifications will be: use of clinically available serum calcium, if not available, ionized calcium could be used for safety monitoring purposes with less than 0.3 mL of blood. The investigators

could also eliminate the VDBP assay in this infant (as this is an exploratory aim) to reduce the amount of blood needed. 25(OH)D on the volume of blood remaining after a CBC is run, this option could be investigated to reduce blood draw volume.

E. Describe briefly the statistical methods used to analyze the data. *See Educational Guide – Biomedical Research (PedsIRB)*

The primary endpoint for statistical analysis is serum 25(OH)D level at discharge. Analysis will be done using intent-to-treat strategy, where infants will be included in the group to which they were assigned regardless of compliance. The statistical analyses will be done with SAS software. P-values less than O.O5 will be considered statistically significant. Baseline characteristics (e.g. gender, season of birth) will be compared between the dose groups, stratified by race and gestational age, to ensure comparability of the groups using Mann-Whitney tests for continuous variables and Fisher's exact tests for categorical variables. Analysis of covariance (ANCOVA) will be conducted to test for differences in discharge serum 25(OH)D level between the dose groups while adjusting for known confounders (race, gestational age, gender and season of birth). If the overall F-tests are significant then pairwise comparisons will be made and p-values for these comparisons will be adjusted for multiple comparisons using Tukey's method. Correlation methods will be used to look at associations between 25(OH)D levels and iPTH levels at discharge. Descriptive plots will be used to evaluate changes in serum 25(OH)D levels. DEXA scan results evaluating total body minus head bone mineral density (BMD) will be compared between the two groups. Descriptive plots will be used to evaluate changes in serum 25(OH)D levels. All tests will be 2-sided and p-values <0.05 will be considered statistically significant.

F. Does this research involve genetic testing?

☒ **No**

☐ **Yes. Attach *Addendum G.*** *See Educational Guide – Biomedical Research (PedsIRB)*

G. After completion of this research, will any un-used HBM be used to create a tissue bank for future research? *See Educational Guide – Biomedical Research (PedsIRB)*

☒ **No.**

☐ **Yes. Respond to the following:**

1) What is the location of the tissue bank?

2) Where will the HBM be stored and how will it be secured?

3) Who will have access to the HBM and what is the process for obtaining samples from the bank?

4) How will confidentiality be protected when HBM is released from the tissue bank?

5) What records will be kept regarding disposition of samples?

6) What is the procedure for a subject to withdraw his/her HBM?

7) Will any clinical data be obtained and banked with the HBM?

☐ **No**

☐ **Yes. Respond to the following:**

a) Specify the source of the clinical data (e.g., medical records).

b) **Specify what clinical data will be collected.**

c) **Indicate the HBM donor identifiers that will be recorded from the medical, or other record, and maintained (at any time) in association with the HBM.** *See Educational Guide – Biomedical Research (PedsIRB)*

- ☐ **Names**
- ☐ **Postal address information: street address, city, county, precinct, ZIP code**
- ☐ **All elements of dates (except year) related to an individual (e.g. birth, admission, discharge).**
- ☐ **Telephone numbers**
- ☐ **Fax numbers.**
- ☐ **Electronic mail addresses**
- ☐ **Social Security numbers**
- ☐ **Medical Record numbers**
- ☐ **Health plan beneficiary numbers**
- ☒ **Account numbers**
- ☐ **Certificate/license numbers**
- ☐ **Vehicle identifiers and serial numbers, including license plate numbers**
- ☐ **Device identifiers and serial numbers**
- ☐ **Web Universal Resource Locators (URLs)**
- ☐ **Internet Protocol (IP) address numbers**
- ☐ **Biometric identifiers, including finger and voice prints**
- ☐ **Full face photographic images [and any comparable images]**
- ☐ **Any other unique identifying number, characteristic, or code**

OR

- ☐ **No HBM donor identifiers will be recorded and maintained in association with the HBM.**

12. DRUGS AND BIOLOGICS

A. Does this study involve an investigational drug or biologic?

- ☒ **No**
- ☐ **Yes. Respond to the following:**

1) **Identify the investigational drug or biologic.**

2) **Provide the IND number and holder of the IND. If the IND is held by the PI, provide a copy of the FDA notification letter, or other verification and copies of the signed investigator statements (Form FDA 1572) for all required study personnel in accordance with 21 CFR 312.53.** *See Educational Guide – Biomedical Research (PedsIRB)*

3) **Describe the controls for securely storing and dispensing the investigational drug or biologic in both the in-patient and out-patient setting.** *See Educational Guide – Biomedical Research (PedsIRB)*

B. Does this study involve the investigational use of an FDA-approved and marketed

drug or biologic? *See Educational Guide – Biomedical Research (PedsIRB)*

☐ **No**

☒ **Yes. Respond to the following:**

1) **Does the drug or biologic have an IND number?**

   ☒ **No**

   ☐ **Yes. Provide the IND number and identify the holder.**

2) **Will the results of this study be used by the manufacturer to support (a) a new indication for the drug or biologic, (b) a significant change in the labeling for the drug or biologic, or (c) a significant change in the advertising for the drug or biologic?**

   ☒ **No**

   ☐ **Yes. Explain.** *See Educational Guide – Biomedical Research (PedsIRB)*

3) **Does the study involve a route of administration or dosage level, or use in a subject population or other factor that significantly increases the risks (or decreases the acceptability of the risks) associated with the use of the drug or biologic?**

   ☒ **No**

   ☐ **Yes. Explain.** *See Educational Guide – Biomedical Research (PedsIRB)*

13. FDA USE-IN-PREGNANCY CATEGORY

Provide an FDA Use-in-Pregnancy category (A, B, C, D or X) for each drug used as a part of this research. *See Educational Guide – Biomedical Research (PedsIRB)*

This study will administer Vitamin D3 to infants. Vitamin D3 is pregnancy category C. This recommendation is based on animal studies which have shown fetal abnormalities in several species associated with hypervitaminoisis D similar to supravalvular aortic stenosis syndrome described in infants. Hollis et al have done several studies with high dose vitamin D in both infants and pregnant women without adverse events.

14. DEVICES

A. Does this study involve an investigational device?

☒ **No**

☐ **Yes. Respond to the following:**

1) **Identify and describe the investigational device.**

2) **Indicate the sponsor's determination of whether the device is significant risk (SR) or non-significant risk (NSR).**

3) **Provide the IDE number and identify the holder of the IDE for SR devices. If the IDE is held by the PI provide a copy of the FDA notification letter or other verification.** *See Educational Guide – Biomedical Research (PedsIRB)*

4) **Describe controls for securely storing and dispensing the device.** *See Educational Guide – Biomedical Research (PedsIRB)*

5) **Describe oversight procedures in place for departments, sections, or operating rooms where devices are used which provide for independent monitoring of the storage and dispensing of devices as well as required record-keeping.** *See Educational Guide – Biomedical Research (PedsIRB)*

B. **Does this study involve the investigational use of an FDA-approved, marketed device?**
☒ **No**
☐ **Yes. Does the device have an IDE number?**
☐ **No**
☐ **Yes. Provide the IDE number and identify the holder.** *See Educational Guide – Biomedical Research (PedsIRB)*

15. Confidentiality and Privacy

A. **Where will research data be stored and how will it be secured?** *See Educational Guide – Biomedical Research (PedsIRB)*

Electronic data will be protected in accordance with UNMC Computer Use and Electronic Information Security Policy No 6051. All electronic devices are located in locked offices and are password locked and encrypted. Data will be entered and stored on the UNMC secured server hard-drive in order to further protect from unauthorized access and loss of research data.

Hard copies, including Parental consent forms will be stored separately in a locked drawer in a locked office.

B. **Will any of the following subject identifiers be obtained from the medical record and/or directly from the subject or their LAR and maintained (at any time) in association with the research data?** *See Educational Guide – Biomedical Research (PedsIRB)*
☐ **No**
☒ **Yes. Respond to the following:**

1) **Indicate the subject identifiers that will be recorded:**
☒ **Name**
☐ **Postal address information: street address, city, county, precinct, ZIP code**
☐ **All elements of dates (except year) related to an individual (e.g. birth, admission, discharge)**
☐ **Telephone numbers**
☐ **Fax numbers.**
☐ **Electronic mail addresses**
☐ **Social Security numbers**
☒ **Medical Record numbers**
☐ **Health plan beneficiary numbers**
☐ **Account numbers**
☐ **Certificate/license numbers**
☐ **Vehicle identifiers and serial numbers, including license plate numbers**
☐ **Device identifiers and serial numbers**
☐ **Web Universal Resource Locators (URLs)**
☐ **Internet Protocol (IP) address numbers**
☐ **Biometric identifiers, including finger and voice prints**
☐ **Full face photographic images [and any comparable images]**
☐ **Any other unique identifying number, characteristic, or code]**

2) **What is the justification for recording the specific identifiers listed above?**

See Educational Guide – Biomedical Research (PedsIRB)
The identifiers listed above will be maintained will the infant is hospitalized in the NICU. Data will be collected and entered into a database while the infants is hospitalized. After the infant is discharged, the name and medical record number will be deleted from the database and the data will be assigned a subject number. Blood samples that are sent to Creighton for analysis will be de-identified and sent with the subject number.

3) **How long will the subject identifiers be maintained in association with the research data?**
Until the infant is discharged from the hospital and the outpatient DEXA scan has been completed.

C. Will research data that contains subject identifiers be disclosed to anyone at UNMC, TNMC, UNO or CHMC who is <u>not</u> listed in Section I of this application?

☒ **No**
☐ **Yes. Identify by name.** *See Educational Guide – Biomedical Research (PedsIRB)*

D. Will research data that contains subject identifiers be disclosed to any investigators <u>outside</u> of UNMC, TNMC, UNO or CHMC?

☒ **No**
☐ **Yes. Respond to the following:**

1) **Identify the investigator by name and affiliation.**

2) **Specify the subject identifiers which will be associated with the data.**

3) **Explain the necessity for this disclosure.** *See Educational Guide – Biomedical Research (PedsIRB)*

E. Will research data that contains subject identifiers be disclosed to any commercial sponsor, contract research organization (CRO) or Data and Safety Monitoring Board (DSMB)?

☒ **No**
☐ **Yes. Respond to the following:**

1) **Identify by name and affiliation.**

2) **Specify the subject identifiers which will be associated with the data.**

3) **Explain the necessity for this disclosure.** *See Educational Guide – Biomedical Research (PedsIRB)*

F. Will research data that contains subject identifiers be disclosed to any other external organization or entity (e.g., NCI cooperative groups)?

☒ **No**
☐ **Yes. Identify by name.**

G. Will research data be shared with third party payers?

☒ **No**

☐ **Yes.** *See Educational Guide – Biomedical Research (PedsIRB)*

H. For what duration of time will research data be subject to disclosure to the persons or groups identified above? *See Educational Guide – Biomedical Research (PedsIRB)*

I. What provisions will be in place to protect the subject's privacy? *See Educational Guide – Biomedical Research (PedsIRB)*

Consent will be obtained in the patient's individual private room. Unauthorized personnel will not be involved in consenting patients and will not have access to patient identifiers for the purposes of the study. Specific subject identifiers will not be directly associated with the research data. The patients will be referred to as de-identified numerical subjects

J. Does this research involve data banking at UNMC, TNMC, UNO or CHMC for future use or for purposes that are not integral to the current research?

☒ **No**

☐ **Yes. Complete and attach *Addendum I*.**

RISK/BENEFIT ASSESSMENT (16-22)

16. POTENTIAL RISKS

What are the potential risks associated with each research intervention? If data are available, estimate the probability that a given harm may occur. *See Educational Guide – Biomedical Research (PedsIRB)*

This study will be presented as a greater than minimal risk for physical and psychological harm.

Supplemental vitamin D3 400 IU or 800 IU will be given to the respective groups. Hypercalcemia has been associated with supplementation resulting in serum levels of above 150 ng/mL. Based on preliminary data, we do not expect our supplementation protocol to achieve 25(OH)D levels of >30 ng/mL. We would not expect this dose of vitamin D to carry any potential risk to the subject, in fact this dosing is consistent with an American Academy of Pediatrics guideline that recommends all term, healthy infants consuming less than 1000 mL of formula a day receive 400 IU vitamin D3 supplementation, and the European Society for Gastroenterology, Hepatology, and Nutrition recommendation that premature infants receive 1000 IU/day of vitamin D3 (18).

Hypersensitivity to vitamin D resulting in hypercalcemia can occur in individuals with primary hyperparathyroidism. Infants with this condition would be excluded from the study.

Study subjects would also have increased blood draws for study purposes compared to infants not enrolled in the study. Each subject would have approximately 4 mL of blood drawn over the course of 8 weeks (2 mL per 4 weeks) that a non-study infant would not be exposed to. Increased phlebotomy can result in anemia and risk of infection. Many blood draws are for routine care in the Intensive Care Nursery, so blood for study purposes would be drawn at the same time as patient care labs whenever possible to avoid a painful procedure for the infant. Collection of a urine sample represents minimal risk to an infant, there is a small risk of skin breakdown which is minimized by changing the collection bag every 24 hours.

DEXA systems assess body composition by measuring the differential absorption of x-rays at two frequencies and can separate tissue into fat, lean, and bone mineral. DEXA is relatively easy to administer and uses a very low level of radiation, approximately 1/6 to 1/2 the exposure from a standard chest x-ray. At doses much higher than these infants will receive, radiation is known to increase the risk of developing cancer after many years. At the doses these infants will receive, it is very likely that we will see no effects at all.

All of the proposed risks and benefits will be presented to the mother of the infant using oral and printed information prior to a request for an informed consent to participate in the study.

17. RISK CLASSIFICATION

What is the overall risk classification of the research?

☐ **Minimal risk**
☒ **Greater than minimal risk**
☐ **Significant risk**
See Educational Guide – Biomedical Research (PedsIRB)

18. MINIMIZATION OF RISK

A. Will the research utilize procedures in order to obtain data which will be performed on the subjects in the course of their normal clinical care (i.e. for diagnostic or treatment purposes)?

☐ **No**
☐ **Yes. Describe the procedures.** *See Educational Guide – Biomedical Research (PedsIRB)*

The blood obtained at birth will be from the cord blood that is routinely drawn by the labor and delivery units at TNMC and stored at the patient's bedside in the NICU.

B. Describe how potential risks will be minimized and how the subjects of the research will be monitored by the investigators and other research personnel to ensure their safety. *See Educational Guide – Biomedical Research (PedsIRB)*

Blood is drawn at regular and frequent intervals for patient management during an infant's intensive care nursery stay. Blood for study purposes is drawn at the same time as routine patient care labs whenever possible. Developmentally appropriate nursing interventions such as swaddling, pacifier use, or Sweet-Ease are used at the discretion of the nurse during lab draws to minimize the infant's discomfort.

Urine collection bags will be changed with every diaper change.

C. Is there is a DSMB for this study?

☒ **No. Complete question 18d below.**
☐ **Yes. Submit the DSMB charter or respond to the following information:**

1) Membership composition

2) The authority of the DSM.

3) The timing of the DSMB meeting

4) The frequency of DSMB reports. *See Educational Guide – Biomedical Research (PedsIRB)*

D. If there is no DSMB assigned to this study, describe the data monitoring plan. Specifically:

1) Who will perform the ongoing data and safety analysis?

The PIs will monitor the data on an ongoing basis for any instances of hypercalcemia or hypervitaminosis D. Subjects will be withdrawn from the study should any abnormalities in calcium or vitamin D metabolism occur.

Hypercalcemia (as defined by a serum calcium as ≥12.5 mg/dL or an ionized calcium of 1.35) will be used as the main measure of toxicity, as hypercalcemia is the primary adverse event associated with vitamin D toxicity. These values occur in real time.

25(OH)D levels will also be monitored for 25(OH)D levels above 150 ng/mL. Blood for 25(OH)D levels will be transported to the Creighton Osteoporosis Research Center (CUORC) for analysis. This blood is analyzed when the CUORC has enough samples to fill out a run of 40 samples, including any other samples they may be analyzing. Therefore, in our previous experience, blood samples for 25(OH)D levels occurred more than once a month.

2) What is the frequency of data analysis?
See Educational Guide – Biomedical Research (PedsIRB).

E. Describe the specific <u>subject withdrawal</u> criteria. If *none,* provide an explanation. *See Educational Guide – Biomedical Research (PedsIRB)*
A subject will be withdrawn from the study at the request of the parent or attending neonatologist. A subject would be withdrawn from the study with the diagnosis of GI, liver, or kidney disease, inborn errors of metabolism, parathyroid disease, disorders of calcium metabolism, and any condition requiring treatment with seizure mediation or steroids. A subject could also be withdrawn at the request of the parent or guardian. Ongoing monitoring of the data will allow for any subject with lab abnormalities related to vitamin D or calcium to be withdrawn from the study. A subject would be withdrawn with a serum calcium above 12.5 mg/dl after initiation of the vitamin D, or a 25(OH)D level above 150 ng/mL.

F. Describe the specific <u>stopping rules for the research</u>. If *none*, provide an explanation.
See Educational Guide – Biomedical Research (PedsIRB).
The study would be stopped in the event that even a small amount of the infants enrolled in the study (>5 infants) developed hypercalcemia (serum calcium above 12.5, ionized calcium about 1.35) or hypervitaminosis D (25(OH)D levels above 150 ng/mL).

G. Describe the auditing plan for the research (i.e., who will conduct the audit and how often). If *none,* provide an explanation. *See Educational Guide – Biomedical Research (PedsIRB)*
None

19. POTENTIAL BENEFITS TO THE SUBJECT
What are the anticipated benefits (if any) to the subjects that may reasonably be expected from participation in the research? *See Educational Guide – Biomedical Research (PedsIRB).*
We anticipate subjects participating in the research will have increased 25(OH)D levels over non-participants. This may be associated with improved bone mineralization and an improved resistance to infection.

20. POTENTIAL BENEFITS TO SOCIETY
What are the anticipated benefits (i.e., value) to society that may reasonably be expected to result from this research? *See Educational Guide – Biomedical Research (PedsIRB)*
Suboptimal vitamin D levels are associated with poor bone health and increased incidence of respiratory tract infections, both of which are areas of high concern in a population of infants born prematurely or hospitalized. The results of this project will be of great interest to health care practitioners caring for infants, especially preterm infants, and could affect routine vitamin D supplementation practices. This research will add to the body of knowledge available regarding optimal 25(OH)D levels in this population. This research is novel because it investigates the relationship of VDBP in a population where very little is known.

21. RISK-BENEFIT RELATIONSHIP OF THE RESEARCH

What is the risk-benefit relationship of the research? *See Educational Guide – Biomedical Research (PedsIRB)*
The risks associated with this level of vitamin D supplementation are low, and the benefits of achieving optimal 25(OH)D levels are becoming more clearly defined. We feel the benefits of increasing 25(OH)D levels in this population outweigh the potential risks from supplementation.

22. ALTERNATIVES TO PARTICIPATION

A. How does the study treatment or intervention differ from the care that the subject might receive were he/she not to participate in the research? *See Educational Guide – Biomedical Research (PedsIRB)*
Subjects not enrolled in the research will not receive more than 400 IU of supplemental vitamin D. Supplementation of vitamin D above 400 IU is not standard practice in this population at this time.

B. Are there other reasonable alternative procedures or courses of treatment available in the non-research context at CHMC and/or UNMC/TNMC, or at other institutions, which may be of reasonable benefit to the prospective subject? *See Educational Guide – Biomedical Research (PedsIRB)*
No

C. Would any of the study procedures or courses of treatment in the protocol be available to the prospective subject if they elected not to participate?
☒ **Yes. Explain.**
A subject could receive vitamin D supplementation if ordered by the attending neonatologist.

☐ **No. Explain.** *See Educational Guide – Biomedical Research (PedsIRB)*

D. How do the risks of the research compare with the risks of alternative procedures or courses of treatment described above?
The alternative procedure to participation in the study would be the initiation of vitamin D supplementation by the attending neonatologist. The risks of supplementation would be the same in either case.

E. How do the anticipated benefits of the research compare with the benefit of alternative procedures or courses of treatment described above? *See Educational Guide – Biomedical Research (PedsIRB)*
The alternative procedure to participation in the study would be the initiation of vitamin D supplementation by the attending neonatologist. The benefits of supplementation would be the same in either case. In this case, however, the effect of the supplementation on serum levels of 25(OH)D or iPTH would not be routinely assessed. 25(OH)D levels could be orders, however as a hospital lab these would be run on a mass spectrometer, which has been shown to overestimate 25(OH)D levels in infants less than 1 year of age. This could lead to the assumption of adequacy in deficient infants, or concerns regarding oversupplementation in other.

FINANCIAL OBLIGATIONS AND COMPENSATION (23-24)

23. FINANCIAL OBLIGATIONS OF THE SUBJECT

A. Will sponsors, third party payors, including Medicare, or the Institution cover the

costs of the protocol related treatments and procedures. *See Educational Guide – Biomedical Research (PedsIRB)*
Cost of the protocol will be covered by the Institution

B. **What financial obligations will the parent(s)/guardians of the subject incur as a result of participating in the study?** *See Educational Guide – Biomedical Research (PedsIRB)*
None

C. **Will the financial obligations of the parent(s)/guardians of the subject be increased as a result of procedures performed solely for research purposes?**
☒ **No**
☐ **Yes. Provide additional detail and justification for charging the parent(s)/guardians of the subject for these procedures.** *See Educational Guide – Biomedical Research (PedsIRB)*

24. **COMPENSATION TO THE SUBJECT FOR PARTICIPATION**
Will the subject receive any compensation for participation?
☒ **No**
☐ **Yes. Describe the form of compensation, dollar amount (if applicable) and the prorated compensation plan (if applicable).** *See Educational Guide – Biomedical Research (PedsIRB)*

PRIOR REVIEW (25)

25. **PRIOR IRB REVIEW**
A. **Has this study (or one substantially similar) been previously submitted to the PedsIRB (or the PedsIRB or the UNMC IRB) and then withdrawn by the investigator for any reason?**
☒ **No**
☐ **Yes. Respond to the following:**
1) **Describe why the study was withdrawn.**

2) **Describe changes made to the research plan prior to the current submission.**

B. **To the best of your knowledge, has this study (or one substantially similar) been considered by another IRB and not granted approval?**
☒ **No**
☐ **Yes. Provide details of the disapproval including the name of the reviewing IRB, date of review, and reasons for disapproval.**

SUBJECT IDENTIFICATION, RECRUITMENT AND CONSENT/ASSENT (26-33)

26. **METHOD OF SUBJECT IDENTIFICATION AND RECRUITMENT**
A. **How will prospective subjects be identified (e.g., from a clinic population, hospital inpatient units, previous research participants, support groups, databases)?**
Subjects will be identified from the NICU, a hospital inpatient unit.

The study will also be listed on clinicaltrials.gov where it may be viewed by physicians and patients may learn about the study through that mechanism.

B. Does the PI, secondary investigator(s) or participating personnel have ethical access to the names of prospective subjects?

☐ **No. Describe how these names will be obtained.**

☒ **Yes. Describe the ethical access.** *See Educational Guide – Biomedical Research (PedsIRB)*

Dr. Anderson is the medical director of the NICU and has ethical access to all patient information. Dr. Hanson provides training and coverage to NICU hospitalized patients. Dr. Hanson recently trained a Medical Nutrition Therapist, Melissa Thoene, to provide routine coverage to NICU patients. Laura Dugick, APRN is an NNP in the NICU at TNMC. Dr. Anderson, Laura, and Melissa will screen potential patients for inclusion and approach the parents for permission to discuss the study. If the parents agree, Dr. Anderson or Dr. Hanson will proceed with the consent process.

C. How will prospective subjects be recruited (e.g., personal contact, advertisements, fliers)? *See Educational Guide – Biomedical Research (PedsIRB)*

Infants will be screened upon admission to the NICU and the parents/guardians of those who meet eligibility criteria will be approached regarding enrollment. No advertisements or notices are planned

D. What efforts will be made to achieve appropriate study population diversity? *See Educational Guide – Biomedical Research (PedsIRB)*

Every effort will be made to achieve appropriate study population diversity. It is our expectation that enrollment will be evenly distributed among gender and racial/ethnic distribution of individuals who fulfill eligibility criteria. We expect our population diversity to reflect the population diversity of the NICU. We do not expect withdrawals from the study as the study will take place in an inpatient setting and has a very low risk profile. If we notice any refusal of consent within any ethnic or racial groups, every effort will be made to determine what the barriers to enrollment are, and to improve enrollment in these groups.

27. PROCESS OF PERMISSION (INFORMED CONSENT) FOR PARENT OR GUARDIAN

A. When will the parent(s) or guardian(s) of the prospective subject be approached relative to their child's actual participation in the study? *See Educational Guide – Biomedical Research (PedsIRB)*

Infants will be screened upon admission to the NICU by investigators with ethical access to the patient information (Dr. Anderson and Melissa Thoene). After receiving permission to discuss the study with them, parents of eligible infants will be approached for enrollment in the study by Dr. Hanson or Dr. Anderson. A detailed explanation of the research will be given, including basic study design and the risks and benefits. An interpreter will be utilized as needed if any language barriers exist, and parents will be provided with a fully translated consent form (for Spanish-speaking parents).

B. What is the location where informed consent will be obtained, and how will the environment be conducive to discussion and thoughtful consideration by the parent/guardian? *See Educational Guide – Biomedical Research (PedsIRB)*

The NICU has consultation rooms and a library that is quiet and private, and will allow for a conversation in a stress-free, quiet, and private setting. Adequate time will be taken to answer all questions from the parents/guardian regarding the study. Delayed consent will be used to allow time for any discussion regarding enrollment.

C. Who will be involved in the process of consenting the parent(s) or guardian(s) about their child's participation in the study and what are their responsibilities? *See*

Educational Guide – Biomedical Research (PedsIRB)
The principal and secondary investigators, as well as participating personnel, will be involved in the process of consent. The investigators and the participating personnel will all have an in-depth enough understanding of the study to discuss the risks, benefits, and design of the study. Dr. Hanson and Dr. Anderson will be available to answer any detailed questions if contacted by study personnel, or at the request of the parents.

D. How much time will be allotted to the process of consent? *See Educational Guide – Biomedical Research (PedsIRB)*
As much time as is needed will be allotted for the process of consent. Study personnel will remain to answer questions and to assure that parents understand the study. Potential subjects will be allowed to take the consent form home before signing. Potential subjects will be re-approached within 72 hours after the initial contact to obtain delayed consent

E. How will the process of consent be structured for parent(s) or guardian(s) who are likely to be more vulnerable to coercion or undue influence? *See Educational Guide – Biomedical Research (PedsIRB)*
One of the following methods will be used for individuals who are likely to be vulnerable, based on the situation: The family and friends of the parent/subject will be involved, or an advocate for the parent/subject will be appointed, or reading the consent out loud to the parent/family/advocate

F. How will consent be obtained from non-English speaking parent(s) or guardian(s)? *See Educational Guide – Biomedical Research (PedsIRB)*
Hospital interpreters will be utilized in this process if language barriers exist

G. How will it be determined that the parent(s) or guardian(s) understood the information presented? *See Educational Guide – Biomedical Research (PedsIRB)*
A "teach-back" method will be utilized to assess understanding of the consent process. Parents will be asked to state back to the study personnel key concepts of the informed consent.

H. Will there be a formal process of on-going re-consent (over and above re-consent associated with changes in protocol)?
☐ **No**
☐ **Yes. Describe.** *See Educational Guide – Biomedical Research (PedsIRB)*

28. PROCESS OF OBTAINING ASSENT FROM THE CHILD

A. Will the investigator ask children and adolescents to assent to participate in the research?
☐ **Yes**
☒ **No. Complete and attach *Addendum L.*** *See Educational Guide – Biomedical Research (PedsIRB)*

B. How will it be determined that the child understood the information presented? *See Educational Guide – Biomedical Research (PedsIRB)*

C. Will children reach the age of majority (i.e., 19 years old) during the course of the study?
☒ **No**
☐ **Yes. Describe the process of re-consent.** *See Educational Guide – Biomedical Research (PedsIRB)*

29. INFORMATION PURPOSELY WITHHELD

Will any information be purposely withheld from the parent(s)/guardian of the subject or the subject during the research or after completion of the research?

☒ **No**

☐ **Yes. Respond to the following:**

A. What specific information will be withheld?

B. What is the justification for this non-disclosure?

C. Will information that has been withheld eventually be shared with the subject and/or parent?

☐ **No. Provide justification**

☐ **Yes. When and how will information be shared (e.g., after completion of his/her participation in the research or after completion of the research project in its entirety)?** *See Educational Guide – Biomedical Research (PedsIRB)*

30. DOCUMENTATION OF CONSENT AND ASSENT

Identify, by name, the investigator(s) and participating personnel who will document obtainment of informed consent from the parent(s)/guardian and assent from the subject.
See Educational Guide – Biomedical Research (PedsIRB)

The following investigators and participating health care personnel who will obtain informed consent for the study protocol are listed below. These individuals have the clinical expertise and sufficient knowledge about the protocol and IRB consent requirements. All of the participating health care personnel are CITI trained and will be trained on the protocol of the study.

Corrine Hanson, PhD, RD
Ann Anderson Berry, MD

31. CONSENT AND ASSENT FORMS

Indicate the type of consent forms and study information sheets to be used in this research:

☒ **Parental consent form**
☐ **Youth Study Information Sheet**
☐ **Child Study Information Sheet**
☐ **Screening consent form**
☐ **Addendum consent form**
☐ **Adult consent form**
☐ **Other (***See Educational Guide – Biomedical Research (PedsIRB)*

32. WAIVER OR ALTERATION OF INFORMED CONSENT

Is a waiver or alteration of consent requested?

☒ **No**

☐ **Yes. Complete** ***Addendum J.*** *See Educational Guide – Biomedical Research (PedsIRB)*

33. WAIVER OF A SIGNED CONSENT FORM

Is a waiver of the requirement to obtain a signed consent form requested?

☒ **No**
☐ **Yes. Complete *Addendum M.*** *See Educational Guide – Biomedical Research (PedsIRB)*

RESOURCES (34)

34. Describe the resources available to safely conduct this study at all sites specified in Section I.8. *See Educational Guide – Biomedical Research (PedsIRB)*

The Clinical Research Center at the University of Nebraska Medical Center offers support for research projects, including nursing support, minority community consultation and support, research pharmacy support, and research laboratory analysis.

Dr. Hanson has access to a private computer in a locked office. This computer is supported by UNMC and can safely store research data in secure servers. Statistical analysis will be done by Ms. Lyden in the College of Public Health. The College of Public Health provides hardware and software

to meet all the College's computing needs (including database and statistical) without having to employ outside resources. The College's facilities include:

- Local Area Network (LAN) using Microsoft Server 2000 network operating system. The LAN runs on a Dell network file server providing 150GB of RAID5 storage. Remote server backup is performed nightly, with all backup media stored in a secure location off site.

- Network-attached Intel Pentium based workstations (using Windows XP operating systems) for all statisticians, faculty, programmers, analysts, and clerks.

- Network-attached Hewlett Packard laser printers and Dell color laser printer accessible from all network workstations.

- Networked FAX capability via a Xerox 657 Document center. The system provides Atraditional@ FAX services, as well as allowing users to send faxes directly from their desktop.

Networked microcomputers are used for all College computing activities. These include data entry, database management, data analysis, word processing, presentation development, geographic information system (GIS) applications, and other office practice activities. In addition to standard office application software, a wide variety of analytic software is employed by College personnel, including SAS, SPSS, S-Plus, Gauss, LirnDep, SUDAAN, and ArcView. Finally, a variety of notebook computers are owned by the College and provided for faculty/staff use.

Our study is strengthened by our ability to work with a DEQUA certified laboratory at the Creighton Osteoporosis Research Center to obtain an accurate estimation of the 25(OH)D status of this population. Dr. Hanson, Dr. Anderson, Dr. Armas, and Ms. Lyden have previously collaborated and have gained considerable experience with vitamin D and neonatal research. VDBP will be analyzed in the laboratory of Dr. Stephen Rennard. Dr. Rennards lab is very experienced with VDBP assay and Dr. Rennard has more than 30 years of experience designing ELISA assays, which will be used to quantify VDBP. Taken together, the overall experience of these investigators strongly supports the feasibility of the proposed work to further our understanding of vitamin D status of NICU hospitalized infants to begin to lay the groundwork for vitamin D's pivotal role in infant health.

CLINICAL TRIAL REGISTRY (35)

35. Will this study be listed in a clinical trial registry (e.g., http://ClinicalTrials.gov/)?

☐ **No**

☒ **Yes. Respond to the following:**

A. Provide the name of the registry and web address if different than www.ClinicalTrials.gov.

B. Provide the registration number for this trial. *See Educational Guide – Biomedical Research (PedsIRB)*

Registration at clinicaltrials.gov is in progress

LITERATURE REVIEW (36)

36. REFERENCES

Provide a full listing of the key references cited in the background (Section II.2). The references should clearly support the stated purpose of the study. *See Educational Guide – Biomedical Research (PedsIRB)*

1. Kovacs C. Vitamin D in pregnancy and lactation: maternal, fetal, and neonatal outcomes from human and animal studies. *Am J Clin Nutr.* 2008;88(2):520S-528S.

2. Bodnar LM, Simhan HN, Powers RW, Frank MP, Cooperstein E, Roberts JM. High prevalence of vitamin D insufficiency in black and white pregnant women residing in the northern United States and their neonates. *J Nutr. 2007;137(2):447-452.*

3. Saintonge S, Bang H, Gerber LM. Implications of a new definition of vitamin D deficiency in a multiracial us adolescent population: the National Health and Nutrition Examination Survey III. *Pediatrics.* 2009;123(3):797-803.

4. Ginde AA, Liu MC, Camargo CA,Jr. Demographic differences and trends of vitamin D insufficiency in the US population, 1988-2004. *Arch Intern Med.* 2009; 169(6):626-632.

5. Nesby-O'Dell S, Scanlon KS, Cogswell ME, et al. Hypovitaminosis D prevalence and determinants among African American and white women of reproductive age: third National Health and Nutrition Examination Survey, 1988-1994. *Am J Clin Nutr. 2002;76(1):187-192.*

6. Mahon P, Harvey N, Crozier S, et al. Low Maternal Vitamin D Status and Fetal Bone Development: Cohort Study. *J Bone Miner Res. 2009.*

7. Yorifuji J, Yorifuji T, Tachibana K, et al. Craniotabes in normal newborns: the earliest sign of subclinical vitamin D deficiency. *J Clin Endocrinol Metab. 2008;93(5):1784-1788.*

8. Weiler H, Fitzpatrick-Wong S, Veitch R, et al. Vitamin D deficiency and whole-body and femur bone mass relative to weight in healthy newborns. *CMAJ.* 2005; 172(6):757-761.

9. Zamora SA, Rizzoli R, Belli ~C, Siosman DO, Bonjour JP. Vitamin D supplementation during infancy is associated with higher bone mineral mass in prepubertal girls. *J Clin Endocrinol Metab.* 1999;84(12):4541-4544.

10. Javaid MK, Crozier SR, Harvey NC, et al. Maternal vitamin D status during pregnancy and childhood bone mass at age 9 years: a longitudinal study. *Lancet. 2006;367(9504):36-43.*

11. Gordon CM, Feldman HA, Sinclair L, et al. Prevalence of vitamin D deficiency among healthy infants and toddlers. *Arch Pediatr Adolesc Med. 2008;162(6):505-512.*

12. Gordon CM, OePeter KC, Feldman HA, Grace E, Emans SJ. Prevalence of vitamin D deficiency among healthy adolescents. *Arch Pediatr Adolesc Med. 2004;158(6):531-537.*

13. Harkness L, Cromer B. Low levels of 25-hydroxy vitamin D are associated with elevated parathyroid hormone in healthy adolescent females. *Osteoporos Int.* 2005; 16(1): 1 09-113.

14. Sullivan SS, Rosen CJ, Halteman WA, Chen TC, Holick MF. Adolescent girls in Maine are at risk for vitamin D insufficiency. *JAm Diet Assoc. 2005;105(6):971-974.*

15. Wayse V, Yousafzai A, Mogale K, Filteau S. Association of subclinical vitamin D deficiency with severe acute lower respiratory infection in Indian children under 5 y. *Eur J Clin Nutr.* 2004;58(4):563-567.

16. Cannell JJ, Vieth R, Umhau JC, et al. Epidemic influenza and vitamin D. *Epidemiolinfeci.* 2006; 134(6): 1129-1140.

17. Walker V, Xiaoran Z, Rastergar I, Liu P, Hollis B, Adams J, et al. Cord Blood Vitamin D Status Impacts Innate Immune Responses. *J Clin Endocrin Melab.* 2011 ;96(6):1-9.

18. Agostoni C, Buonocore G, Carnielle V, De Curtis M, Darmaun, D, Desci, T. et al. Enteral Nutrient Supply for Preterm Infants: commentary From the European Society for Paediatric Gastroenterology, Hepatology, and Nutrition Committee on Nutriton. *JPGN.* 2010;50(1):85-91.

SECTION III

SUBMISSION DEADLINE
Note: Incomplete submissions may result in delay of IRB review

A. **Full Board Review:** The IRB meets monthly, on the fourth Tuesday of the month. No more than 15 applications (i.e., initial review of a new study, and re-review of a tabled study will be reviewed at each meeting. All reviews are performed on a first-come first-served basis. The IRB meeting schedule and deadline dates can be found on the IRB website at www.unmc.edu/irb.

B. **Expedited Review:** Applications that qualify for expedited review have no submission deadline and can be reviewed independent of the IRB meeting schedule. Please call the Office of Regulatory Affairs for assistance in determining if your study meets the requirements for expedited review.

SUBMISSION CHECKLIST

A. ***Number of Copies/Packets:***
- ☐ Original "packet" printed single-sided and paper-clipped.
- ☐ **26** "packets" (Materials in each packet must be printed consecutively front-to-back and the each packet is stapled together as one item.) *Note: If this study qualifies for expedited review only **1** copy is required.*

B. ***Each "packet" must contain the following items in this specific order:***
- ☐ Pediatric Biomedical Research Application
- ☐ Parent/Guardian and/or adult informed consent form(s)
- ☐ Youth and/or Child Study Information Sheet(s)
- ☐ Subject recruitment material
- ☐ ***For studies conducted at UNMC or TNMC*:** Pharmacy and Therapeutics (P&T) Committee Investigational Drug Study Registry and/or Marketed Drug Form
- ☐ ***For studies conducted at CHMC:*** *(Studies performed solely at CHMC will be exempt from review by the TNMC Pharmacy and Therapeutics Committee provided relevant drug information is submitted and reviewed as required by CHMC policy 204.0).*
- ☐ Performance site approval for all non-UNMC, TNMC, UNO and CHMC sites
- ☐ Copy of all questionnaires, surveys, assessment tools, and other relevant materials
- ☐ Clinical Trial Master Matrix as required by UNMC and TNMC.

C. ***5 copies of each of the following documents (separate from the packet), copied separately front-to-back and stapled:*** *Note: If this study qualifies for expedited review only **1** copy of each document is required.*
- ☐ Detailed protocol
- ☐ Investigator's brochure
- ☐ Grant Application

D. ***1 copy of the following forms (as applicable):***
- ☐ *IRB Review Fee Form* for all commercially sponsored research projects.
- ☐ *UNMC Disclosure of Potential Conflict of Interest Form* for the Principal Investigator if a financial interest has been declared in Section I.11. *Note: A COI management plan for the PI conflict of interest must be developed before this application will be reviewed by the IRB*
- ☐ *UNMC Disclosure of Potential Conflict of Interest Form* for any responsible personnel with a

financial interest declared in Section I.11. *Note: Completed UNMC Disclosure of Potential Conflict of Interest Forms must contain all required signatures. Send completed forms to the Office of Regulatory Affairs (UNMC – 7680). The ORA will forward the forms to the UNMC Compliance Officer for review.*

ADDITIONAL REVIEW REQUIREMENTS

Final IRB approval and release of studies is contingent upon approval by the following UNMC committees or departments. Check the appropriate boxes:

- ☐ ***Pharmacy and Therapeutics (P&T) Committee:*** Review by the P&T Committee is required for all protocols involving the use of investigational or marketed drugs. *Note: Protocols conducted solely at CHMC will be exempt from review by the TNMC Pharmacy and Therapeutics Committee provided relevant drug information is submitted and reviewed as required by CHMC Policy.*
- ☐ ***UNMC Eppley Cancer Center Scientific Review Committee (SRC):*** Review by the SRC is required for all protocols involving cancer patients conducted at UNMC or TNMC. Protocols conducted solely at CHMC, when the PI is a member of the Cancer Center, also require review by the SRC. Protocols conducted solely at CHMC when the PI is not a member of the Cancer Center are exempt from SRC review.
- ☐ ***Institutional Biosafety Committee (IBC):*** Review by the IBC is required for all protocols involving the use of gene transfer and vaccines.
- ☐ ***Radioactive Drug Research Committee (RDRC):*** Review by the RDRC is required for all protocols involving the use of a radio-labeled drug for which the investigator or the institution holds the IND.
- ☐ ***Sponsored Programs Administration (SPA)/Office of Regulatory Affairs:*** For commercial sponsored studies, the consent form and contract will be compared for consistency. Final IRB approval and release is contingent upon completion of a signed contract for all commercially sponsored research.
- ☐ ***Conflict of Interest Committee (COIC):*** All responsible personnel listed in Section I of the IRB application (i.e., PI, Secondary Investigator, Participating Personnel, and Protocol Coordinator) must disclose **any** financial interest in the research. Data and Administrative Personnel are exempt. The COIC will review any financial interest which is classified as significant.

  A disclosure of financial interest in the research requires submission of the *UNMC Disclosure of Potential Conflict of Interest Form* (available on the UNMC Conflict of Interest website at http://www.unmc.edu/dept/compliance/index.cfm?L1_ID=32&CONREF=8). This form must be submitted with this IRB application or separately.

  Note: Responsible personnel, as listed above, are considered covered persons. Any financial interest related to the research accruing to the immediate family, including parent(s), or spouse of a parent, spouse, dependent child of a covered person, or anyone that a covered person may claim as a dependent under the Internal Revenue Code will be treated as a financial interest of the covered person which, in turn, will require a disclosure of financial interest.

  Note: The existence of a financial conflict of interest is an important consideration in the IRB's review. In some cases, a management plan to minimize the conflict is necessary. If such a management plan is needed, it could range from disclosure of the conflict in the consent document, to exclusion of an investigator from obtaining consent, to disqualification of an investigator. It is the obligation of the PI to ensure that all responsible personnel listed for this study have complied with the above described disclosure requirements.
